# From nanotechnology to immunomodulation: emerging strategies targeting macrophages in high altitude pulmonary hypertension

**DOI:** 10.1186/s12951-026-04317-1

**Published:** 2026-04-03

**Authors:** Yi Zhu, Xiuli Yang, Xiyu Cao, Ke Liu, Kexin Yu, Jianli Ma, Junru Zhang, Kaijie Dang, Ke Chen, Kun Qian, Xiaobo Wang, Qihang Ding, Zhen Cheng, Chuantao Zhang

**Affiliations:** 1 Department of Respiratory Medicine, Hospital of Chengdu University of Traditional Chinese Medicine, Chengdu, 610072 Sichuan China; 2 School of Clinical Medicine, Innovative Institute of Chinese Medicine and Pharmacy/Institute of Interdisciplinary Studies, Chengdu University of Traditional Chinese Medicine, Chengdu, 610075 Sichuan China; 3 Department of Chemistry, Korea University, Seoul, 02841 Republic of Korea; 4 State Key Laboratory of Drug Research, Molecular Imaging Center, Shanghai Institute of Materia Medica, Chinese Academy of Sciences, Shanghai, 201203 China; 5 Shandong Laboratory of Yantai Drug Discovery, Bohai Rim Advanced Research Institute for Drug Discovery, Yantai, 264117 Shandong China

**Keywords:** High altitude pulmonary hypertension, Nanoplatform, Macrophage-targeted therapy, Pulmonary vascular remodeling, Immunotherapy.

## Abstract

High altitude pulmonary hypertension (HAPH) is a public health issue in high-altitude regions, characterized by increased pulmonary artery pressure, pulmonary vascular remodeling, and right heart failure, leading to the high incidence rate and high mortality. Current therapies can alleviate patient symptoms but provide limited control over disease progression. Immune regulation has gained recognition as a central driver of HAPH. Among immune cells, macrophages, as key immune regulators in the lung, orchestrate HAPH pathogenesis through recruitment, M1/M2 polarization, inflammatory activation, metabolic reprogramming, and crosstalk with pulmonary vascular cells. Targeting macrophages has emerged as a promising therapeutic strategy. Nanoplatforms provide powerful tools to enable precise targeting and functional modulation of macrophages in HAPH, as nanoplatforms such as liposomes, polymeric nanoparticles, and exosomes enable targeted delivery and controlled release, thereby facilitating precise regulation of the pulmonary immune microenvironment, including both macrophages and pulmonary vascular cells. Recent advances in nanoplatforms have enabled the development of macrophage focused precision interventions. In this review, we first outline the pathogenic roles of macrophages in the development and progression of HAPH. We then summarize the therapeutic potential of nanoplatform-based approaches in HAPH, with a particular focus on emerging nanomaterial-based strategies that enable targeted modulation of macrophage function. We argue that nanoplatform assisted macrophage immunotherapy holds strong potential to move HAPH treatment from symptomatic management toward mechanistic and precision interventions.

**Graphical abstract:**

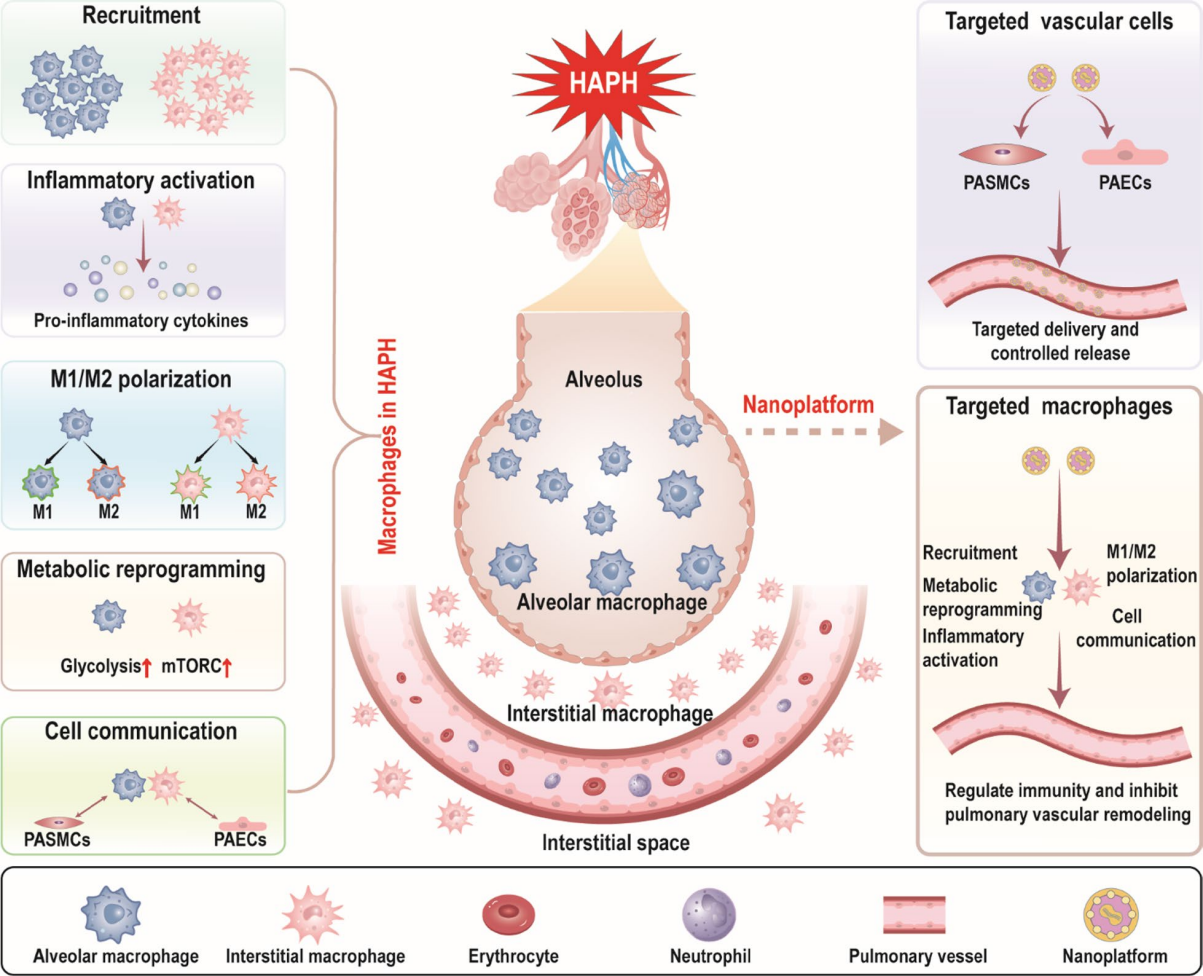

## Introduction

High altitude pulmonary hypertension (HAPH) is a public health issue in high-altitude regions, characterized by increased pulmonary artery pressure, pulmonary vascular remodeling, and right heart failure, leading to a high incidence rate and high mortality [1, 2]. Long-term exposure to a low-oxygen environment at high altitudes is the predominant cause of HAPH [3]. Worldwide, more than 80 to 120 million people live at an altitude of at least 2500 m (accounting for more than 1% of the total population), China has the largest absolute population at an altitude above 3500 m [4]. Among people at high altitudes, the prevalence of HAPH can be as high as 35% [5]. Therefore, HAPH has become a public health problem in high-altitude areas around the world [6]. Although the pathophysiological understanding of pulmonary hypertension has improved over the past decade, the treatment methods are still limited, vasodilation therapy (phosphodiesterase inhibitors, carbonic anhydrase inhibitors, endothelin receptor blockers) can temporarily relieve symptoms. However, their control effect on disease progression is limited, and they are accompanied by side effects and high treatment costs. The best treatment for HAPH is to move to a lower-altitude area. Unfortunately, due to socio-economic reasons, this may not be feasible for many patients, even after successful lung transplantation, the median survival period of patients was only 6.2 years [3, 6, 7]. Consequently, understanding HAPH pathogenesis and developing precise treatment methods have become urgent research priorities.

Nanotechnology offers unique advantages in treating disease [8]. This platform offers a novel and precise therapeutic strategy for HAPH, featuring a multifunctional system capable of targeted drug delivery, high resolution imaging and integrated theranostics [9–11]. As a flexible and adaptable platform [12], nanotechnology enables simultaneous targeting of multiple pathological processes in the pulmonary vasculature, providing a foundation for multimodal and precision medicine approaches in HAPH.

Recent advances have substantially improved our understanding of HAPH. Growing evidence suggests that the immune system, particularly macrophages, plays a pivotal role in the pathogenesis and progression of pulmonary hypertension, including HAPH [13–16]. As one of the most abundant resident immune cells in the lungs, macrophages are markedly recruited under hypoxic conditions [17–19]. These cells undergo substantial phenotypic, functional, and metabolic alterations [20], including M1/M2 polarization, inflammatory activation, and metabolic reprogramming, while engaging in extensive crosstalk with neighboring cells [20–23]. As a result, these macrophage driven processes contribute to pulmonary vascular remodeling and elevated pulmonary arterial pressure.

Given the central role of macrophages in HAPH pathogenesis, they have emerged as promising therapeutic targets [17, 24]. Preclinical studies have demonstrated that various pharmacological interventions can modulate macrophage function, thereby attenuating HAPH progression [25]. More importantly, with the advent of nanotechnology, nanomaterial-based platforms provide means to selectively modulate macrophage function, integrating targeted delivery with immune regulation, thereby complementing traditional vascular cell-targeted therapies [26, 27]. Although pulmonary hypertension encompasses various subtypes sharing features of hypoxia and vascular remodeling [28], HAPH is distinctively characterized by chronic hypoxia-induced inflammation and medial hypertrophy of distal arterioles, without the severe parenchymal destruction seen in pulmonary hypertension associated with lung disease or the plexiform lesions typical of idiopathic pulmonary arterial hypertension [29, 30]. Furthermore, HAPH is fundamentally different from chronic thromboembolic pulmonary hypertension, as it is not caused by mechanical obstruction from organized thrombi, but rather by maladaptive cellular responses to environmental hypoxia [31]. Crucially, the recruitment of macrophages triggered specifically by the hypoxic microenvironment acts as a primary driver for HAPH progression. Therefore, therapeutic strategies that specifically target the hypoxic macrophage phenotype via nanotechnology offer a precise rationale for HAPH treatment, distinguishing this approach from broad-spectrum vasodilators used in other pulmonary hypertension forms. In this review, we comprehensively summarize the contributions of macrophages to HAPH progression and the therapeutic potential of nanoplatform-based approaches. We further highlight current nanoplatform-assisted macrophage-targeted immunotherapies, emphasizing the potential of combining immunomodulation with precision nanomedicine. This provides a framework for developing innovative and multimodal interventions aimed at improving HAPH therapy and patient outcomes.

## Macrophages as key drivers of HAPH progression

**Fig. 1:**
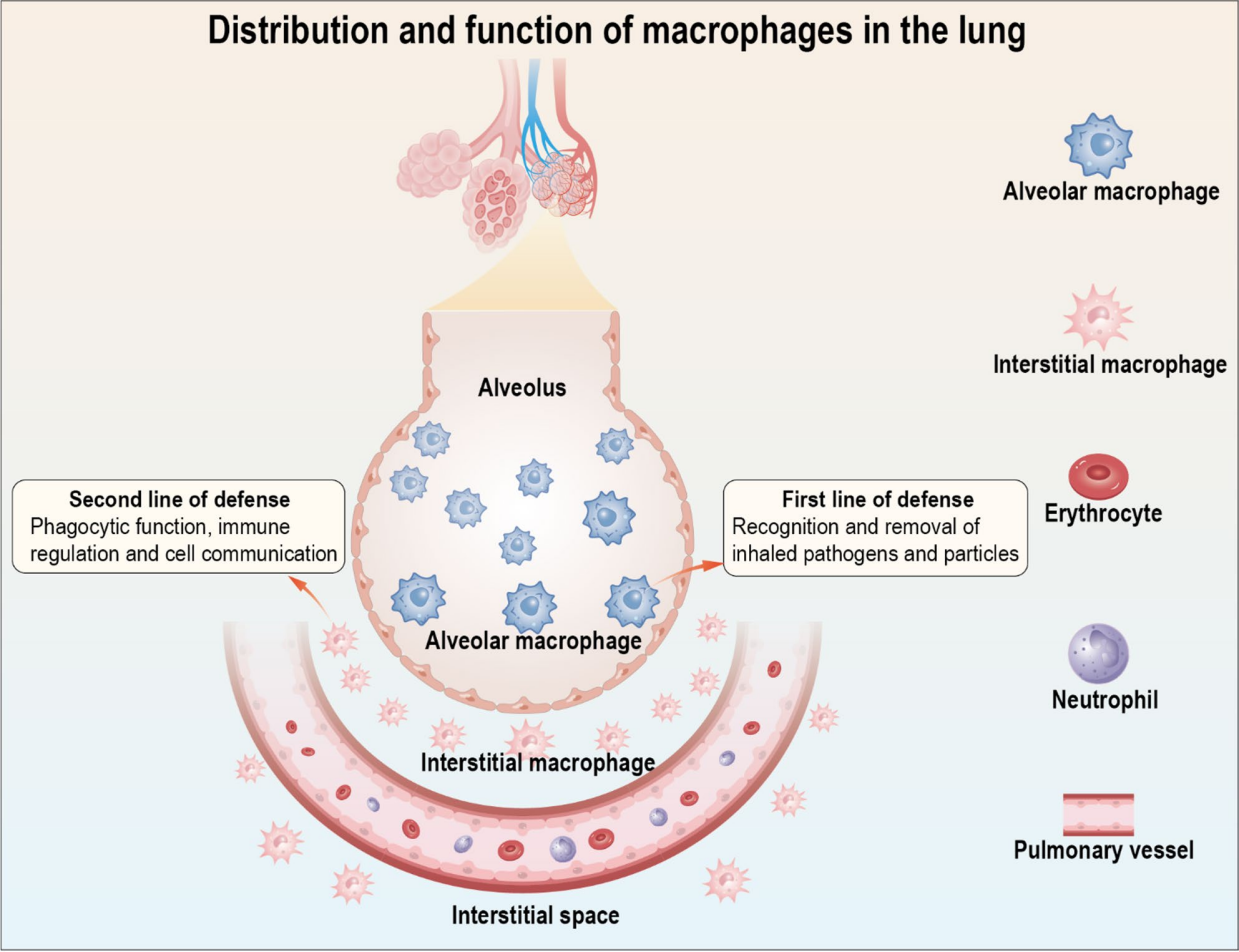
Distribution and function of macrophages in the lung. AMs reside in the alveolar lumen and serve as the first line of defense by clearing inhaled pathogens and debris. IMs are located in the pulmonary interstitium and contribute to immune regulation and cell communication as a secondary defense layer

Macrophages represent one of the most abundant innate immune cell populations in lung tissue [32], these cells play a central role in maintaining pulmonary immune homeostasis, defending against pathogens, and facilitating tissue repair. As key regulators, they orchestrate innate and adaptive immunity, tissue homeostasis, angiogenesis, and metabolic processes [33, 34]. Pulmonary macrophages are primarily classified into two distinct subsets: alveolar macrophages (AMs) and interstitial macrophages (IMs), which exhibit marked differences in anatomical localization, developmental origins, and functional properties [35] (Fig. 1). AMs predominantly reside in the alveolar lumen, closely interacting with type I and II alveolar epithelial cells, capillary endothelial cells, and interstitial fibroblasts [36]. Their ontogeny has been a subject of debate [37], they may originate from yolk sacs and fetal livers during embryonic development [38]. Circulating monocytes may also differentiate into AMs [39], although this contribution is considered minimal [40]. Functionally, AMs serve as the lung’s first line of defense against inhaled pathogens and particulate matter, owing to their potent phagocytic capacity and immunomodulatory activity [34, 37]. Under homeostatic conditions, they maintain alveolar sterility by clearing cellular debris, microbes, and environmental particles while secreting anti-inflammatory mediators to prevent excessive immune activation and tissue damage [34, 41]. AMs exhibit remarkable functional plasticity. During respiratory viral infections, they adopt a pro-inflammatory phenotype, producing cytokines, phagocytizing infected cells, and promoting tissue repair [42]. Similarly, airway epithelial injury can trigger AMs to secrete elevated levels of pro-inflammatory factors [43, 44]. This dual capacity for immune regulation balancing anti-inflammatory homeostasis and pathogen-driven inflammation highlights their pivotal role in pulmonary immunity. IMs are strategically localized in the alveolar septa, perivascular regions, and bronchial walls, where they function as sentinels of the pulmonary vasculature and interstitial compartment [45]. Their origin includes both embryonically derived macrophages and monocyte derived populations recruited during inflammation [46]. Compared with AMs, IMs’ regulatory mechanism and homeostatic function are poorly understood [47]. Nevertheless, emerging evidence suggests their role as a secondary defense barrier. Following bacterial challenge, IMs expand and upregulate interleukin (IL)-10 production, indicative of an anti-inflammatory role [48]. Since IMs are close to non-hematopoietic cells, it is reasonable to assume that there is crosstalk communication between IMs and adjacent cells, although these mediators remain largely unknown [34]. Macrophages possess a high degree of heterogeneity and plasticity. When the lungs are exposed to endogenous or exogenous stress signals, macrophages exhibit functional and phenotypic specialization, effectively respond to environmental signals, and rapidly alter phenotypes and physiological functions [34, 35].

Macrophages contribute to the development and progression of HAPH through a series of phenotypic and metabolic changes. Substantial evidence demonstrates marked macrophage accumulation in pulmonary tissues under hypoxic conditions [49, 50]. During the early phase of hypoxia, macrophages exhibit activation of inflammatory signaling pathways, while in the later stages, pro-remodeling pathways become predominant [51, 52]. Additionally, macrophage polarization is a hallmark feature of HAPH, as evidenced by enhanced M1/M2 phenotypic switching under hypoxic stress [22, 53, 54]. Metabolic reprogramming of macrophages, including changes in glycolytic activity and mitochondrial function, has also been implicated in the pathogenesis of HAPH [20, 55].

## Pathogenic roles of AMs in HAPH

### Recruitment and increase in the number of AMs

Hypoxic exposure triggers AMs recruitment and proliferation, with experimental evidence demonstrating that macrophage depletion attenuates HAPH severity in rodent models [56]. In mice, the recruitment process can be mediated by the upregulation of the chemokine receptor C-C chemokine receptor (CCR) type 5 [57]. Additionally, circulating monocytes may migrate into the alveolar space and differentiate into macrophages, further contributing to the expansion of the AMs population in rats [58]. Thus, the accumulation of AMs represents a crucial initiating mechanism that connects hypoxic stimuli with downstream vascular pathology in HAPH. Importantly, nanomaterials have been shown to directly interact with AMs [59], thereby reducing pro-inflammatory cytokine production and subsequent macrophage recruitment.

### AMs M1/M2 polarization

Under hypoxic exposure, both M1 and M2 macrophage subsets expand in vivo, although their temporal dynamics differ: while M2 macrophages decline after 14 days, they remain persistently elevated relative to baseline. Across multiple murine studies, M2 macrophages consistently emerge as major drivers of pulmonary artery endothelial cells (PAECs) and pulmonary artery smooth muscle cells (PASMCs) dysfunction. For example, M2-derived signals activate signal transducer and activator of transcription (STAT) 3 in PAECs, thereby inducing the IL-33/suppression of tumorigenicity 2 (ST2) axis and promoting vascular remodeling [60], whereas other in vivo studies show that M2 macrophages upregulate the C-X3-C Motif chemokine ligand 1 (CX3CL1)/C-X3-C Motif chemokine receptor 1 (CX3CR1) chemokine axis, directly stimulating PASMCs proliferation [61]. These observations suggest convergence toward a common pro-remodeling phenotype, despite variability in upstream signaling pathways. Importantly, not all studies report identical mechanisms. Hypoxia-induced IL-21 secretion from T follicular helper (Tfh) cells enhances M2 polarization in mice, creating a positive feedback loop that promotes both PASMCs proliferation and pyroptosis [62]. By contrast, rat models emphasize enhanced M2-associated superoxide production, a mechanism closely aligned with oxidative injury [63, 64]. Such interspecies and mechanistic differences highlight the complexity of macrophage-driven remodeling and caution against overgeneralizing findings without considering model-specific contexts. Macrophage M1/M2 polarization also plays a core role in regulating inflammation [65], highlighting it as a potential therapeutic target. In this context, emerging evidence indicates that nanomaterials can modulate AMs polarization, offering a potential strategy to rebalance inflammatory responses [66].

### Inflammatory activation of AMs

Pulmonary macrophage infiltration and excessive inflammatory responses constitute characteristic pathological features of HAPH that actively promote vascular remodeling. Current research has identified multiple signaling pathways responsible for AMs inflammatory activation. Under hypoxic conditions, Pellino E3 ubiquitin-protein ligase 1 (Peli1) expression is upregulated in murine models, where it functions to inhibit forkhead box p1 (Foxp1) expression. This molecular interaction leads to enhanced transcriptional activation of IL-6 and IL-1β, thereby promoting macrophage-mediated inflammatory responses [67]. Concurrently, studies reveal that YTH N6-methyladenosine RNA binding protein 2 (Ythdf2) expression is specifically elevated in AMs under hypoxia. The upregulated Ythdf2 protein exerts its biological effect by restraining heme oxygenase 1 (HMOX1) activity, thereby enhancing the secretion of pro-inflammatory cytokines [68]. Moreover, AMs serve as crucial regulators of pulmonary innate immune homeostasis. Disruption of this regulatory capacity can compromise their protective functions. Specifically, Regnase-1 exerts control over IL-6 and platelet-derived growth factor (PDGF) expression in AMs, thereby preserving lung immune homeostasis and preventing HAPH development in murine models. Under hypoxic conditions, however, downregulation of Regnase-1 expression leads to elevated inflammatory factors. These pro-inflammatory cytokines subsequently stimulate PASMCs proliferation and migration, ultimately driving pulmonary vascular remodeling [69]. Notably, upon differentiation of recruited monocytes into pro-inflammatory AMs in rat lungs, the β3-adrenergic receptor (β3AR)/inducible nitric oxide synthase (iNOS) pathway becomes upregulated. The vasodilatory capacity of iNOS enables inhibition of hypoxic pulmonary vasoconstriction [58]. Overall, these findings indicate that dysregulated inflammatory signaling in AMs contributes to the progression of HAPH by promoting vascular remodeling. IL-6, and PDGF are central regulators linking hypoxic signaling to vascular cell proliferation and migration. These factors not only act directly on PAECs and PASMCs, but also participate in feedback loops that amplify inflammation and vascular remodeling. From a therapeutic perspective, targeting these upstream hubs may yield broader efficacy than focusing on downstream effectors alone. These insights highlight macrophage inflammation as a strategic intervention point. Notably, the inflammatory programs of AMs are closely intertwined with their metabolic states. Recent advances suggest that nanoplatforms can be rationally engineered to target AMs, allowing for selective suppression of inflammatory signaling, offering a novel avenue to attenuate vascular remodeling [70, 71].

### Metabolic reprogramming of AMs

**Fig. 2:**
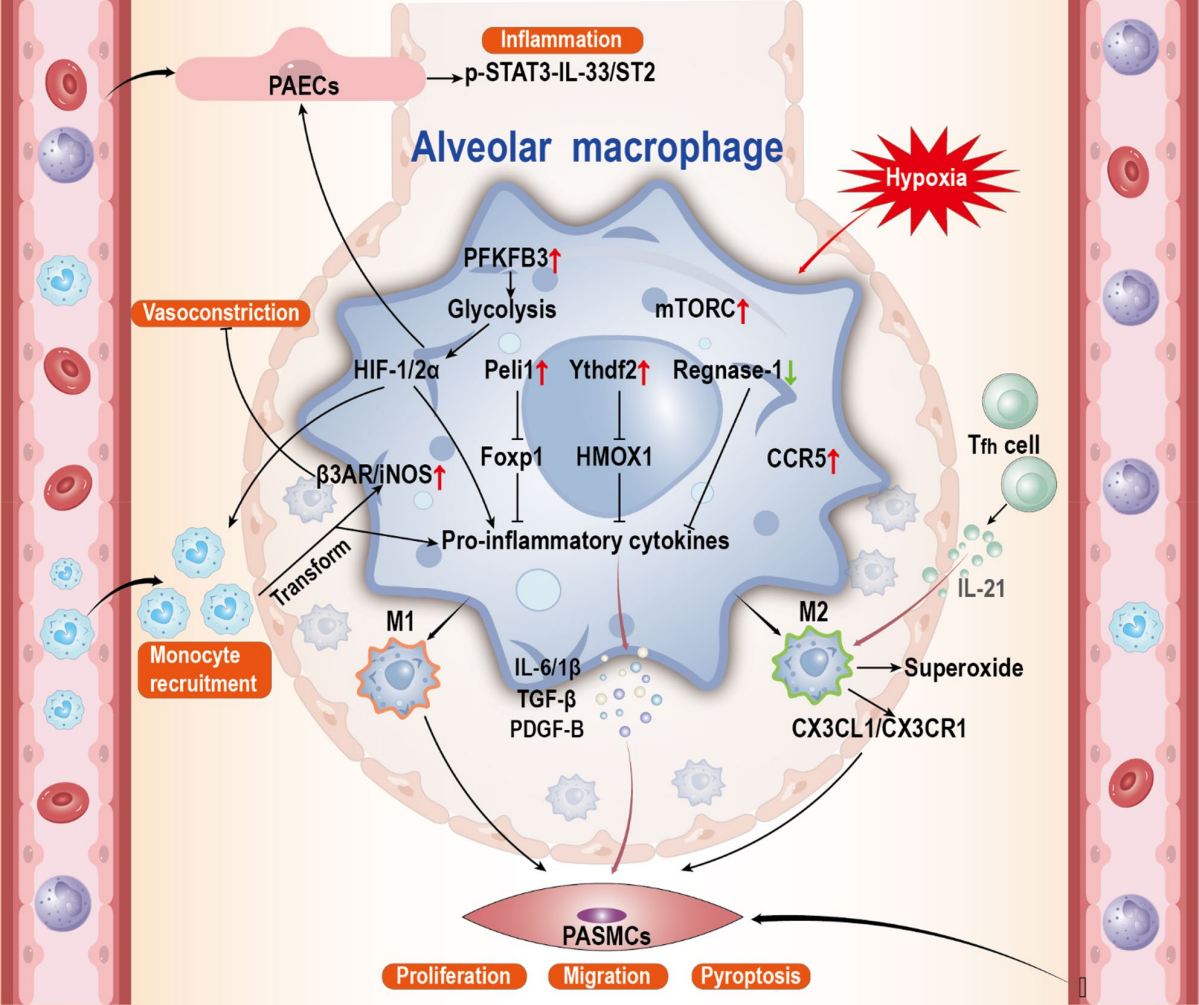
Pathogenic roles of AMs in HAPH. In HAPH, AMs recruit and the functional status of AMs changes, manifested as enhanced metabolic reprogramming, inflammatory activation and M1/M2 polarization, promoting the proliferation, migration and pyroptosis of PASMCs. In addition, AMs interact with various types of cells, such as PAECs, Tfh cells, and monocytes, to synergistically regulate inflammatory responses, vascular tension states, and cell recruitment, jointly promoting the occurrence and development of HAPH

Metabolic reprogramming has emerged as a defining feature of AM responses to hypoxia [72]. Under physiological conditions, AMs rely predominantly on mitochondrial oxidative metabolism and exhibit limited glycolytic dependence, consistent with the glucose-restricted pulmonary environment. However, both in vitro and in vivo studies illustrate that hypoxia induces a marked shift toward glycolysis, largely mediated by upregulation of 6-phosphofructo-2-kinase/fructose-2,6-bisphosphatase 3 (PFKFB3) [73, 74]. Activation of the PFKFB3-glycolysis axis promotes hypoxia-inducible factors (HIF)-1α and HIF-2α stabilization, which in turn enhances macrophage-derived PDGF-B secretion, facilitates monocyte recruitment, and accelerates inflammatory activation of PAECs and proliferation of PASMCs [20, 75]. Parallel evidence indicates that mammalian target of rapamycin (mTOR) complex signaling, a central metabolic hub for immune cell fate and self-renewal, is also upregulated in hypoxic AMs, driving broad transcriptional and metabolic remodeling [76–78] (Fig. 2). Although these findings collectively support a model in which hypoxia-driven metabolic rewiring amplifies macrophage inflammatory programs and vascular remodeling, most available data are derived from rodent models or ex vivo macrophage assays, and direct comparisons across models remain limited. Moreover, the relative contribution of glycolysis versus mTOR-dependent pathways has not been systematically evaluated within the same system, leaving uncertainties regarding their hierarchical or cooperative interactions. Despite these limitations, current evidence converges on a consistent theme, hypoxia consolidates AMs activation through metabolic checkpoints, particularly glycolysis, HIF stabilization, and mTOR signaling, which function as upstream drivers of pulmonary vascular remodeling. These metabolic nodes also present accessible therapeutic entry points. Nanoplatforms designed for AMs targeting can both report metabolic states and selectively modulate glycolytic or mTOR activity, highlighting the potential of integrating metabolic regulation with nanomedicine to attenuate HAPH pathogenesis [79].

## Pathogenic roles of IMs in HAPH

### Recruitment and increase in the number of IMs

The expansion and recruitment of IMs represent hallmark pathological features of HAPH [80, 81]. Compared with AMs, IMs display more dynamic migratory behavior and broader interactions with vascular and stromal cells. Multiple hypoxia-driven signals converge to promote their accumulation.

For example, galectin-3 upregulation in IMs enhances secondary macrophage recruitment and inflammatory activation in rats [82]. Endothelial dysfunction forms a major upstream trigger: hypoxia upregulates vascular endothelial growth factor A (VEGF-A)/vascular endothelial growth factor 2 (VEGFR2) Y949 signaling and vascular permeability, facilitating macrophage extravasation, while reduced endothelial nitric oxide synthase (eNOS) further augments macrophage accumulation in mice [83, 84]. Hypoxia-activated PAECs pathways, including nuclear factor-kappa B (NF-κB) and mitogen-activated protein kinase (MAPK), also strengthen chemotactic cues, and bone morphogenetic protein receptor 2 (BMPR2) deficiency additionally amplifies granulocyte macrophage colony-stimulating factor (GM-CSF) translation, linking genetic susceptibility to enhanced macrophage recruitment [85, 86]. PASMCs contribute additional layers of regulation. Hypoxia induces IL-1R1 and myeloid differentiation primary response 88 (MyD88) expression in PASMCs, promoting macrophage homing to the vessel wall, whereas downregulation of phosphatase and tensin homolog (PTEN) elevates stromal cell-derived factor 1α (SDF-1α) secretion and orchestrates directional macrophage migration in mice [87, 88]. The adventitia also plays an active role, hypoxic pulmonary fibroblasts from hypertensive calves show histone deacetylase (HDAC) activation and C-terminal binding protein 1 (CtBP1)-dependent metabolic reprogramming, along with reduced HMOX1 activity, collectively facilitating macrophage infiltration [89, 90].

Systemic immune components reinforce this process. Platelet activation promotes the secretion of C-X-C motif chemokine ligand (CXCL) 4, colony-stimulating factor 2 (CSF-2), and C-C motif chemokine ligand (CCL) 5 release, while classical dendritic cells generate additional chemotactic stimuli, promoting macrophage accumulation in mice [91, 92]. Under hypoxic conditions, blood monocytes are recruited to the lung perivascular space, where they differentiate into inflammatory macrophages in mice [93]. This process is mediated, at least in part, by HIF-1α upregulation in monocytes [94]. Additionally, downregulation of cytochrome P450 gene Cyp2c44 has been reported to promote hematopoietic stem cell expansion, ultimately driving myeloid differentiation and increasing macrophage production in mice [95]. Collectively, these findings support a model in which IMs recruitment results from coordinated hypoxia-induced signals originating from PAECs, PASMCs, adventitial fibroblasts, and circulating immune cells. However, most mechanisms have been validated only in rodent or large-animal models, and cross-species consistency remains insufficiently evaluated. The relative contribution of each vascular compartment also remains unclear due to heterogeneous experimental designs. These evidence gaps highlight the need for integrated in vivo and human-tissue studies to define dominant pathways. From a translational perspective, IMs represent a tractable target for nanomedicine. Nanoplatforms such as clodronate-loaded liposomes, which effectively deplete pathogenic macrophages in pulmonary fibrosis models [96], illustrate the feasibility of selectively modulating IMs and provide a conceptual basis for macrophage-directed nanotherapies in HAPH.

### M1/M2 polarization and inflammatory activation of IMs

IMs have been increasingly recognized as important contributors to the pathogenesis of HAPH. Similar to AMs, hypoxia drives IMs toward enhanced M1/M2 polarization, which activates downstream signaling in pulmonary vascular cells. For example, enhanced M1/M2 polarization induces p-STAT3 activation in PAECs and subsequently stimulates the IL-33/ST2 axis in mice [60], while M2-type IMs can promote PASMCs proliferation through the CX3CL1/CX3CR1 pathway [61]. Although IMs share functional similarities with AMs, their distinct roles in HAPH require further clarification.

A hallmark of HAPH is the accumulation of lung macrophages around pulmonary vessels, which amplifies perivascular inflammation and vascular remodeling. Single-cell transcriptomic analyses from hypoxic mice have revealed enrichment of interferon-γ, IL-2, and IL-6 signaling in mesenchymal macrophages [51]. Several molecular regulators within macrophages modulate this process. In HAPH rats, downregulation of insulin receptor substrate 2 (IRS2) enhances protein kinase B (AKT) and extracellular signal-regulated kinase (ERK) activation [97]. Similarly, decreased κ-opioid receptor (κ-OR) signaling in macrophages drives IL-6 production, which activates STAT3 and miR-153-3p, ultimately promoting PASMCs proliferation; proliferating PASMCs further suppress κ-OR expression, forming a feedback loop [98]. IL-6 also drives PAECs activation by upregulating endothelin-1 (ET-1) in mice [99]. Additionally, macrophage-derived CCR2/CCR5 promote PASMCs migration and proliferation in vitro [100]. Hypoxia also shapes macrophage inflammatory phenotypes through key upstream mediators. Upregulation of serum glucocorticoid-regulated kinase-1 (SGK1) enhances macrophage secretion of monocyte chemoattractant protein-1 (MCP-1), IL-1β, and tumor necrosis factor-α (TNF-α) in mice [101]. Concurrently, hypoxia-induced activation of TGF-β via thrombospondin-1 (TSP-1) facilitates monocyte recruitment and contributes to vasoconstriction and remodeling [102, 103]. Interestingly, once monocytes accumulate in the lungs of HAPH rats, they differentiate into macrophages that upregulate β3AR/iNOS signaling, leading to vasodilation, suggesting a potential compensatory mechanism [58]. Moreover, macrophages can exert protective roles, in hypoxic mice, breakpoint cluster region (Bcr) and Abr protein upregulation suppresses Rac1 activity, suppresses reactive oxygen species (ROS) generation, and attenuates remodeling [104]. Pulmonary vascular cells themselves actively influence macrophage behavior under hypoxia. Adventitial fibroblasts can activate macrophage inflammatory responses via CtBP1 [105] or through complement-containing small extracellular vesicles in vitro [106]. Their secretion of IL-6 further activates macrophage HIF-1α, CCAAT/enhancer-binding protein β (C/EBPβ)/STAT3, and STAT1 pathways, thereby enhancing vascular remodeling [107]. Additionally, hypoxia-exposed PASMCs downregulate PTEN, resulting in SDF-1α upregulation and IL-6 mediated macrophage activation in mice [88].

Together, these findings position IMs as key regulators of the inflammatory network in HAPH, acting at the interface of vascular and stromal signaling. Inflammatory activation in IMs under hypoxic conditions appears to be orchestrated through a few critical hubs, including IL-6/STAT3 signaling, HIFs. These pathways integrate vascular and stromal cues, positioning IMs as both amplifiers and modulators of the perivascular inflammatory niche. Importantly, these inflammatory activities are metabolically demanding, and hypoxia-induced metabolic reprogramming both fuels and constrains the functional capabilities of IMs, further embedding them within the pathological network of HAPH. Fortunately, several studies have already explored therapeutic strategies targeting IMs inflammation and macrophage polarization. For instance, Chang Liu et al. developed an inhalable hybrid biomimetic nanoplatform to regulate macrophage-driven immune dysregulation in acute lung injury [108], while Jiake Gu et al. designed cerium-luteolin nanocomplexes to modulate inflammatory macrophage responses [109]. These findings highlight the feasibility of nanotechnology-based approaches to precisely reprogram macrophage polarization and alleviate pulmonary inflammation.

### Metabolic reprogramming of IMs

Metabolic reprogramming is a hallmark of macrophage adaptation to hypoxia. Similar to AMs, IMs undergo metabolic shifts mediated by HIFs [110], both HIF-1α and HIF-2α activation drive macrophages to secrete PDGF-B, which in turn induces the proliferation and migration of PASMCs, contributing to vascular pathology in mice [75]. HIFs serve as critical downstream effectors of glycolytic metabolism. Under hypoxic conditions, the upregulation of PFKFB3 in macrophages enhances glycolysis, further stabilizing HIF-1α and HIF-2α. This metabolic shift promotes vascular remodeling through multiple mechanisms, including direct effects on PASMCs, monocyte recruitment, and the exacerbation of inflammatory responses in PAECs in vitro [20]. Additionally, hypoxia-induced mitogenic factor (HIMF) has been identified as a potent activator of HIF-1α, further amplifying its downstream effects in hypoxic macrophages in mice [111].

**Fig. 3:**
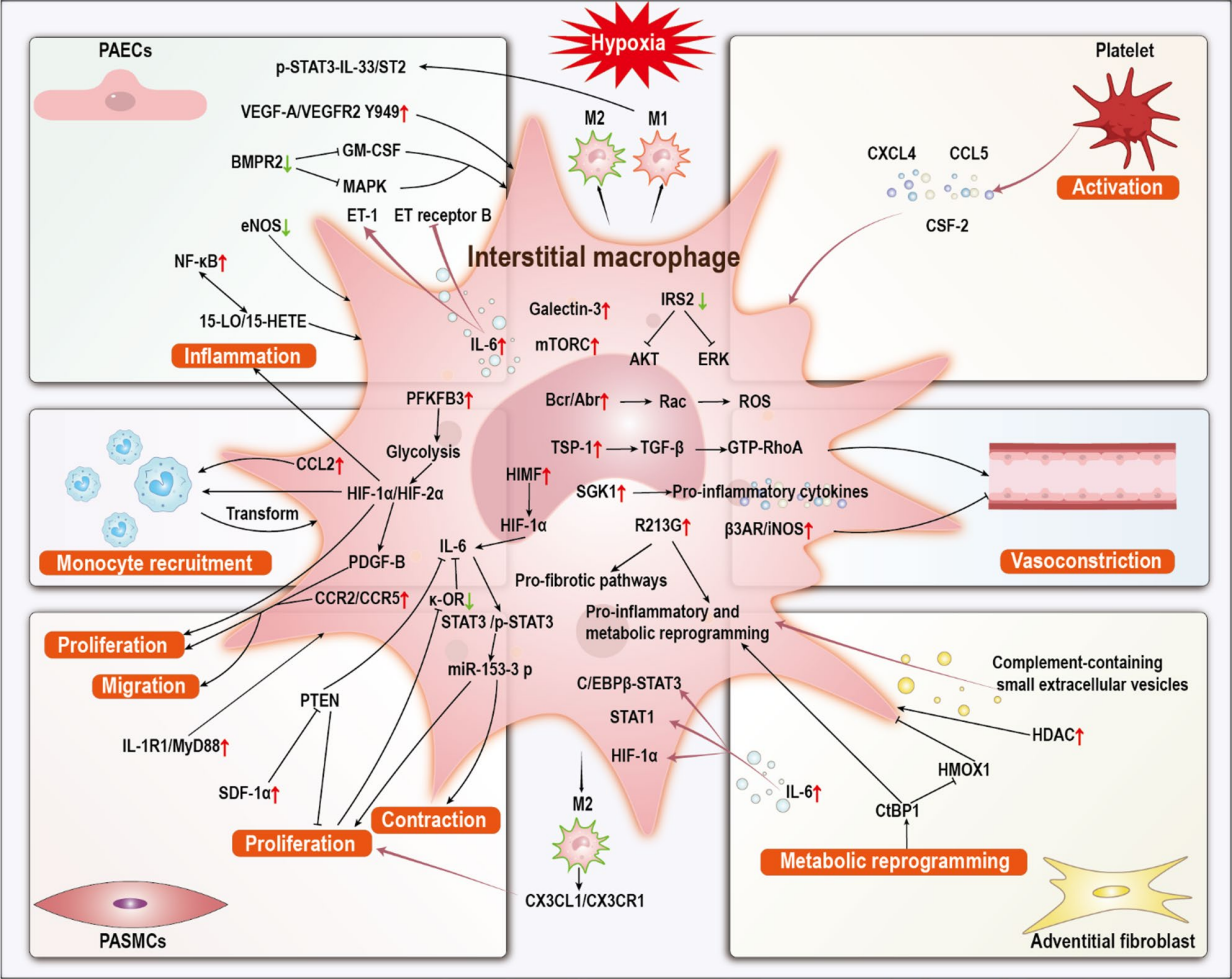
Pathogenic roles of IMs in HAPH. In HAPH, IMs exhibit metabolic reprogramming and phenotypic shaping. The hypoxic environment promotes its transformation to a pro-inflammatory and pro-fibrotic state, manifested as the continuous release of inflammatory factors, proliferation factors and vasoactive molecules, which are involved in the changes of proliferation, migration and contraction functions of PASMCs. Meanwhile, IMs synergically regulate vasoconstriction, matrix remodeling, inflammatory cascade reactions and immune cell recruitment through interactions with various cells such as PAECs, platelets, monocytes and fibroblasts. Furthermore, IMs play a core role in metabolic reprogramming (such as enhanced glycolysis) and the activation of pro-fibrotic pathways

RNA sequencing analyses have elucidated the phenotypic evolution of IMs in HAPH mouse models. At 4 days of hypoxia, IMs exhibit a conserved hypoxia program characterized by mitochondrial dysfunction, activation of pro-inflammatory genes, and mTORC1 signaling [78]. Notably, while the R213G variant did not expand IMs population density in murine models, it induced metabolic rewiring characterized by impaired inflammatory resolution and activation of pro-fibrotic pathways [112]. In addition, the metabolic reprogramming of IMs is further modulated through crosstalk with pulmonary vascular cells, particularly adventitial fibroblasts. These fibroblasts may initiate IMs metabolic shifts through CtBP1 activation [105]. Though the precise signaling mechanisms remain to be fully elucidated. Current evidence suggests potential involvement of complement-containing small extracellular vesicles derived from adventitial fibroblasts in this intercellular communication [106]. Collectively, these findings highlight metabolic reprogramming as a defining feature of IMs under hypoxic stress, enabling their pathogenic transformation and active participation in vascular remodeling. The bidirectional crosstalk between IMs and stromal cells further underscores the microenvironmental regulation of macrophage metabolism in HAPH (Fig. 3). The detailed molecular mechanisms underlying these processes are summarized in Table 1. These metabolic alterations not only define the pathogenic phenotype of IMs but also provide potential therapeutic entry points. Nanoplatforms, with their capacity for precise delivery and functional modulation, offer a promising strategy to specifically target these metabolic pathways in IMs, thereby correcting dysfunction and mitigating HAPH progression.

**Table 1:** The role of macrophages in the pathogenesis of HAPH

Animals	Method	AMs/IMs	Mechanisms	Refs
C57BL/6 mice	10.08% O_2_, 5000 m, 28 d	AMs/IMs	M1/M2 macrophages↑, p-STAT3↑, IL-33↑, ST2↑	[60]
C57BL/6 mice	10% O_2_, 42 d	AMs/IMs	HIF-1α↑, HIF-2α↑, PDGF-B↑	[75]
C57BL/6 mice	10% O_2_, 28 d	AMs/IMs	PFKFB3↑, glycolysis↑, HIF-1α↑, HIF-2α↑	[20]
C57BL/6 mice	5490 m, 28 d	AMs/IMs	mTORC1↑	[78]
C57BL/6 mice	9% O_2_, 18 d	AMs/IMs	CX3CL1↑, CX3CR1↑	[61]
C57BL/6 mice	10% O_2_, 28–42 d	IMs	VEGF-A↑, VEGFR2 Y949↑, IMs↑	[83]
C57BL/6 mice	12% O_2_, 3 d	IMs	CXCL4↑, CCL5↑, CSF-2↑, IMs↑	[91]
C57BL/6 mice	10% O_2_, 8 h/d, 28 d	IMs	κ-OR↓, IL-6↑, STAT3↑, p-STAT3↑, miR-153-3p↑	[98]
C57BL/6 mice	10% O_2_, 4 d or 14 d	IMs	R213G↑	[112]
NC	4570 m, 14 d	IMs	pro-inflammatory and metabolic reprogramming↑	[106]
C57BL/6 mice	10% O_2_, 21 d	IMs	IRS2↓, AKT↑, ERK↑	[97]
NC	4570 m, 14 d	IMs	CtBP1↑	[105]
C57BL/6 mice	5490 m, 4 d			
C57BL/6 mice	10% O_2_, 28 d	IMs	IMs↑, SGK1↑, MCP-1↑, IL-1β↑, TNF-α↑	[101]
C57BL/6 mice	10% O_2_, 21 d	IMs	TSP-1↑, TGF-β↑, GTP-RhoA↑	[102]
SD rats	10% O_2_, 28 d	IMs	Galectin-3↑	[82]
C57BL/6 mice	10% O_2_, 21 d	IMs	HIF-1α↑, IMs migration↑	[110]
NC, C57BL/6 mice	4500 m, 14 d	IMs	CtBP1↑, HMOX1↓, IMs↑	[90]
C57BL/6 mice	5490 m, 4, 14, 28 d			
C57BL/6 mice	9% O_2_, 21 d	IMs	IL-1R1↑, MyD88↑, IMs↑	[87]
C57BL/6 mice	-	IMs	HIMF↑, HIF-1α↑, IL-6↑	[111]
C57BL/6 mice	10% O_2_, 21 d	IMs	IMs infiltration↑	[81]
NC	4500 m, 14 d	IMs	IL-6↑, HIF-1α↑, C/EBPβ↑, STAT3↑, STAT1↑	[107]
WKR	5490 m, 28 d			
C57BL/6 mice	10% O_2_, 21 d	IMs	eNOS↓, IMs↑	[84]
SV129 mice	10% O_2_, 21 d	IMs	IMs↑, IL-6↑, ET-1↑, ET receptor B↓	[99]
SD rats	5000 m, 21 d	IMs	PTEN↓, SDF-1α↑, IL-6↑	[88]
Wistar rats	12% O_2_, 9 d	IMs	NF-κB↑, 15-LO↑, 15-HETE↑, IMs↑	[85]
FVB/J mice	10% O_2_, 21 d	IMs	Bcr↑, Abr↑, Rac1↓, ROS↓	[104]
WKR	5490 m, 28 d	IMs	IMs↑	[80]
NC	4500 m, 14 d			
SD rats	4% O_2_, 8 h/d, 42 d	AMs/IMs	β3AR↑, iNOS↑, CD11c↑, IL-6↑, TNF-α↑	[58]
C57BL/6 mice	10% O_2_, 21 d	IMs	CCL2↑, TSP-1↑, TGF-β1↑	[103]
NC	4500 m, 14 d	IMs	HDAC↑, IMs↑	[89]
WKR	5490 m, 28 d			
C57BL/6 mice	9% O_2_, 21 d	IMs	CCR2↑, CCR5↑	[100]
C57BL/6 mice	10% O_2_, 28 d	AMs	Peli1↑, Foxp1↓, IL-6↑, IL-1β↑	[67]
C57BL/6 mice	10% O_2_, 21 d	AMs	Ythdf2↑, HMOX1↓, IL-6↑, TGF-β↑, PDGF↑	[68]
C57BL/6 mice	10% O_2_, 28 d	AMs	Regnase-1↑	[69]
Wistar rats	10% O_2_, 4 d	AMs	M2 macrophages↑, superoxide production↑	[63]
C57BL/6 mice	10% O_2_, 28 d	AMs	IL-21↑, M2 macrophages↑	[62]
C57BL/6 mice	9% O_2_, 18 d	AMs	CCR5↑, AMs recruitment↑	[57]
SD rats	10% O_2_, 28 d	-	AKT↑	[53]
SD rats	5490 m, 28 d	-	M2 macrophages↑	[13]
SD rats	10% O_2_, 21 d, 28 d	IMs	IMs↑	[18]
C57BL/6 mice	10% O_2_, 35 d	-	G6PD↑, TNF-α↑, M2a macrophages↑	[52]
C57BL/6 mice	10% O_2_, 4 d	-	HIMF↑, hResistin↑, HMGB1↑, RAGE↑	[21]
C57BL/6 mice	10% O_2_, 4 d	-	HIMF↑, IL-4↑, macrophages↑	[19]
C57BL/6 mice	10% O_2_, 14 d	-	IL-6↑, macrophages↑	[49]
C57BL/6 mice	10.5% O_2_, 28 d	-	miR-210↑, ROS↑, macrophages↑	[55]
SD rats	5490 m, 28 d	-	miR-663b↑, AMPK↓, Sirtuin1↓	[54]

AMPK, adenosine monophosphate-activated protein kinase; G6PD, glucose-6-phosphate dehydrogenase; HMGB1, high-mobility group box 1; NC, neonatal calf; WKR, Wistar-Kyoto rats

## Immunotherapy targeting macrophages in HAPH

**Fig. 4:**
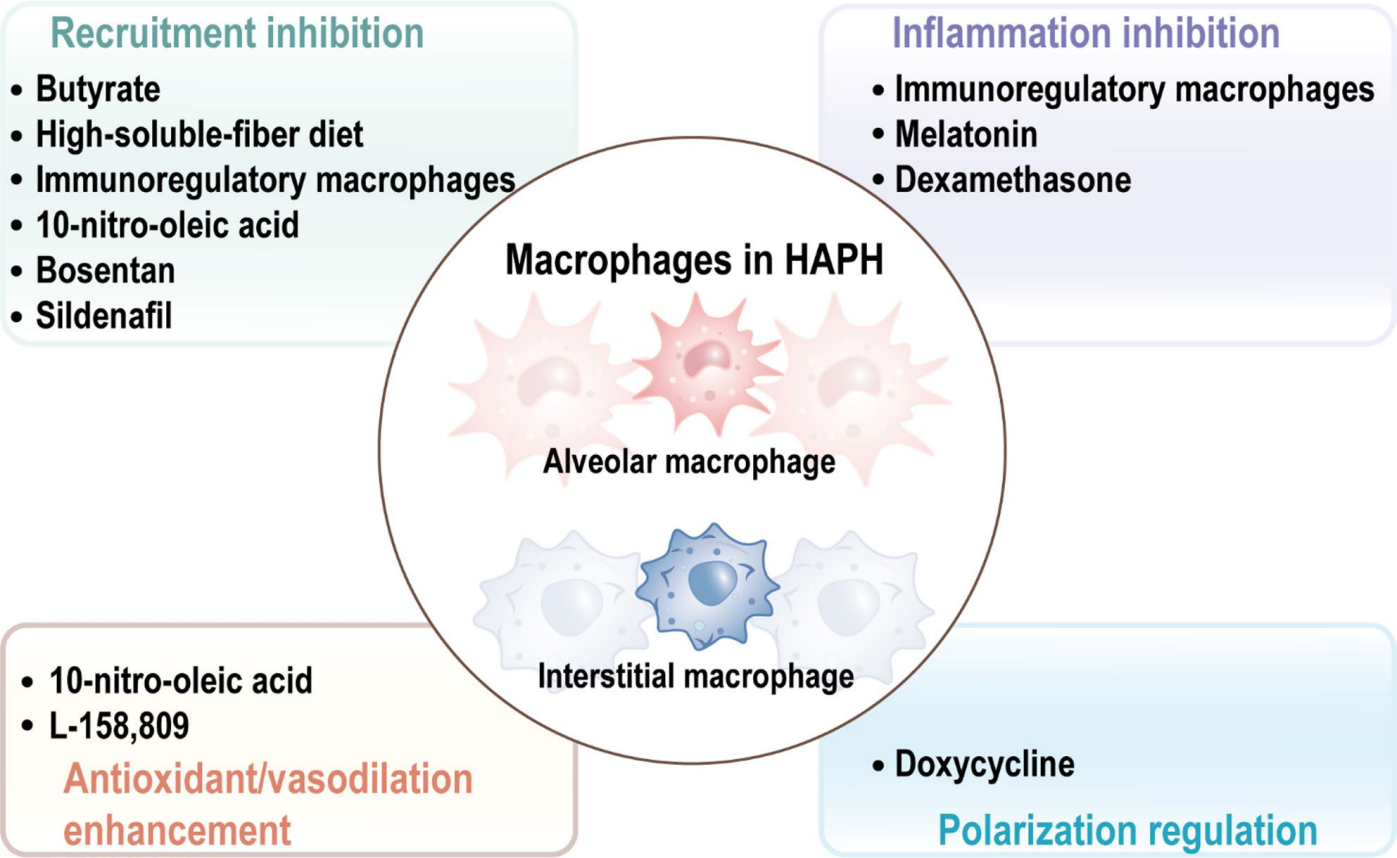
Therapies and mechanisms of macrophage-targeted treatment for HAPH

With the expanding understanding of macrophage involvement in HAPH pathogenesis, numerous therapeutic strategies have emerged targeting pathways related to macrophages (Table 2). Among them, blocking macrophage recruitment and suppressing the activation of macrophage inflammatory pathways are important approaches (Fig. 4). These interventions indicate potential for modulating vascular remodeling processes in HAPH.

Short-chain fatty acids, key microbial metabolites derived from intestinal flora, exhibit therapeutic potential in HAPH. Experimental evidence shows that butyrate administration markedly limits the alveolar and interstitial accumulation of both CD68^+^ and CD163^+^ macrophage populations in hypoxic rats, effectively attenuating inflammation-driven vascular remodeling [113]. Complementary studies reveal that a high-soluble-fiber diet promotes the growth of gut microbiota that produces short-chain fatty acids, elevates circulating propionate levels, and consequently prevents IMs recruitment in HAPH murine models [114]. These findings collectively establish the existence of a functional gut-lung axis in HAPH pathogenesis. Targeting gut microbiota and their metabolic products, particularly short-chain fatty acids, represents a promising macrophage-modulating strategy for HAPH treatment, offering novel therapeutic avenues beyond conventional approaches. The inherent immunomodulatory and antioxidant properties of endogenous hormones offer promising therapeutic potential for HAPH. Melatonin, a pineal neurohormone with well-established antioxidant and anti-inflammatory capacities, displays efficacy in HAPH models. Experimental studies reveal that melatonin treatment attenuates pulmonary inflammation by specifically suppressing inflammasome multiprotein complex formation in AMs, consequently reducing macrophage driven inflammatory cascades in mice [115]. Similarly, dexamethasone, a synthetic glucocorticoid, is a potent anti-inflammatory agent. Dexamethasone has been shown to suppress CCL2 and TSP-1 expression in mice [103]. The nitrergic system is important for regulating vasoconstriction and relaxation, and has crosstalk with macrophage function. 10-nitro-oleic acid attenuated macrophage infiltration and suppressed macrophage superoxide production in mouse models [116]. In addition, by activating the nitrergic system of macrophages, it also helps to dilate blood vessels. The AT(1) receptor blocker L-158,809 induces iNOS activity in IMs in newborn piglets, which causes the blood vessels to dilate [117].

Some unconventional drugs have also shown the potential to treat HAPH by repressing macrophage inflammation. Doxycycline treatment maintained high HMOX1 levels during hypoxia in mice, obstructed M2 macrophage accumulation and activation, and induced macrophage IL-10 expression and mitigated PASMCs hyperproliferation. Ultimately prevent the development of HAPH [118]. Drugs that have been used to treat pulmonary arterial hypertension have also shown targeted macrophage effects, such as bosentan [119] and sildenafil [120], both of them dampened the recruitment of macrophages in hypoxic animal models, suggesting their potential immunomodulatory effects. In addition, cell immunotherapy based on macrophages has also shown an important role in the treatment of vascular remodeling in HAPH. Pulmonary administration modified to macrophages with anti-inflammatory/pro-regression phenotypes can alleviate monocyte recruitment and perivascular inflammation in mice, thus alleviating HAPH [121]. In conclusion, therapeutic strategies targeting macrophages in HAPH represent a rapidly evolving form of immunotherapy. Current interventions can be broadly classified into approaches modulating macrophage recruitment, M1/M2 polarization, inflammatory signaling, and metabolic reprogramming. While these strategies share the common endpoint of attenuating vascular remodeling, their immunomodulatory specificity and durability remain variable, ranging from direct inhibition of chemotaxis to reprogramming transcriptional and metabolic pathways. Moreover, most available evidence derives from hypoxic rodent models, with limited validation in large-animal models or human studies, underscoring the need for translational refinement. Despite these limitations, macrophage-centered immunotherapy highlights the feasibility of reshaping the immune microenvironment as a disease-modifying strategy for HAPH. Yet, a key challenge is tissue and cell-type specificity, since systemic interventions often lack the precision required to selectively modulate pulmonary macrophages. To overcome this, nanoplatforms have emerged as promising enablers, offering enhanced targeting efficiency, stimulus-responsive release, and the ability to integrate drug delivery with gene or gas therapies [122, 123]. Thus, the future of macrophage-targeted immunotherapy lies in the convergence of nanotechnology and immune modulation [124]. Leveraging nanoplatforms to precisely reprogram macrophage recruitment, polarization, and metabolism holds the potential to disrupt the vicious cycle of inflammation and vascular remodeling, paving the way for next-generation precision immunotherapies in HAPH.

**Table 2:** Conventional macrophage-targeted therapy for HAPH

Animals	Therapies	Dose	AMs/IMs	Mechanisms	Refs
SD rats	Butyrate	220, 2200 mg/kg (p.o.) for 14, 28 d	AMs/IMs	Macrophages↓	[113]
C57BL/6 mice	Melatonin	5-8.75 mg/kg (p.o.) for 14 d	AMs/IMs	Inflammasomes↓	[115]
C57BL/6 mice	10-nitro-oleic acid	1.04 nmol/g/h, (s.c.) for 14 d and 28 d	IMs	Macrophages↓, superoxide↓	[116]
Newborn piglets	L-158,809	1 mg/kg (i.p.) for 6 d	IMs	iNOS↑	[117]
C57BL/6 mice	HSF diet	16.9% (p.o.) for 21 d	IMs	Macrophages↓	[114]
C57BL6/ mice	Dexamethasone	1.5 mg/kg (i.p.) for 22 d	IMs	CCL2↓, TSP-1↓	[103]
BALB/c mice	Doxycycline	1 mg/ml (p.o.) for 7 d	AMs	M2 macrophages↓, HMOX1↑, IL-10↑	[118]
C57BL/6 mice	M2_reg_ macrophages	0.6-1 × 10^6^ BMDMs (o.p., i.n.)	AMs	Inflammation↓	[121]
C57BL/6 mice	Bosentan	30 mg/kg (i.p.) for 21 d	-	Macrophages↓	[119]
SD rats	Sildenafil	1.4 mg/kg (i.p.) for 15 d	-	Macrophages recruitment↓	[120]

HSF, high-soluble-fiber

## Nanoplatform-enabled therapeutic approaches for HAPH

Nanotechnology has emerged as a versatile therapeutic platform, enabling targeted delivery, controlled release, and improved bioavailability of therapeutic agents. A wide range of nanomaterials, including lipid-based carriers, exosomes, metal and inorganic nanoparticles, and polymeric nanostructures, have been developed to enhance treatment efficacy while reducing systemic toxicity. These advances provide a foundation for precision intervention in HAPH and set the stage for cell-specific immunomodulatory strategies.

### Natural nanomaterials: exosomes

Among natural nanomaterials, extracellular vesicles, particularly exosomes, have emerged as versatile and biologically compatible drug delivery platforms for the treatment of HAPH. Exosomes are nanoscale vesicles secreted by nearly all cell types and carry bioactive lipids, proteins, and nucleic acids reflective of their parental cells. Their intrinsic biocompatibility, low immunogenicity, and capacity to cross biological barriers confer clear advantages over many synthetic nanocarriers [125, 126]. Importantly, exosomes can be engineered to encapsulate therapeutic cargos, including small molecules, RNAs, and proteins, enabling both endogenous signaling modulation and exogenous drug delivery [127]. These properties position exosomes as promising natural nanoplatforms for targeting macrophage function and pulmonary vascular remodeling in HAPH. Various cell-derived exosomes have been studied, including mesenchymal stromal cell-derived exosomes (MSC-Exos), telocyte-derived exosomes, plasma exosomes, and PAECs-derived exosomes, the mechanistic depth and translational relevance vary substantially across studies.

**Fig. 5:**
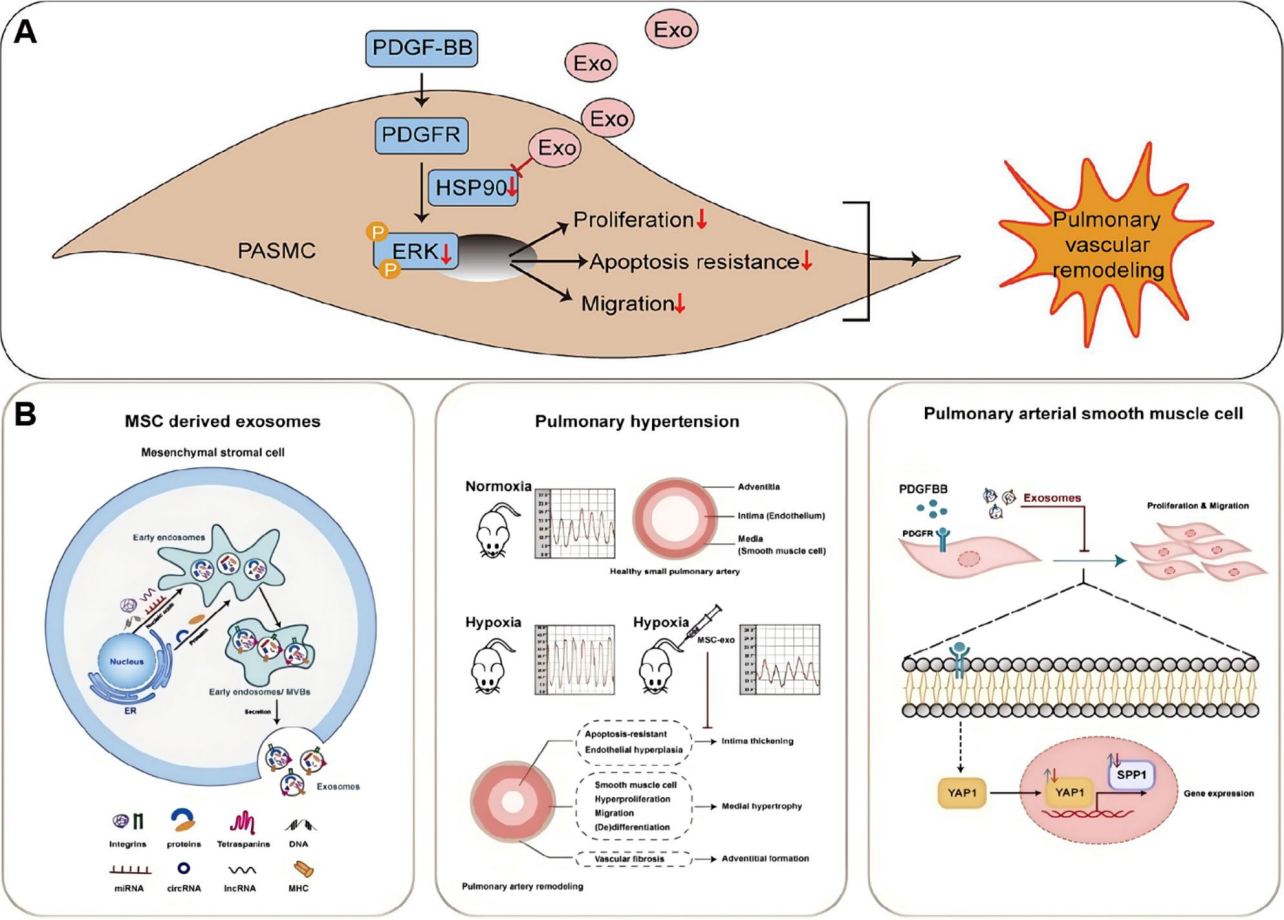
(**A**) MSC-Exos ameliorate hypoxic pulmonary hypertension by inhibiting the Hsp90aa1/ERK/pERK pathway. Reproduced with permission [128]. Copyright. 2023, Elsevier. (**B**) MSC-Exos exert a therapeutic effect against hypoxia-induced pulmonary hypertension by modulating the YAP1/SPP1 pathway. Reproduced with permission [129]. Copyright. 2023, Elsevier

**Fig. 6:**
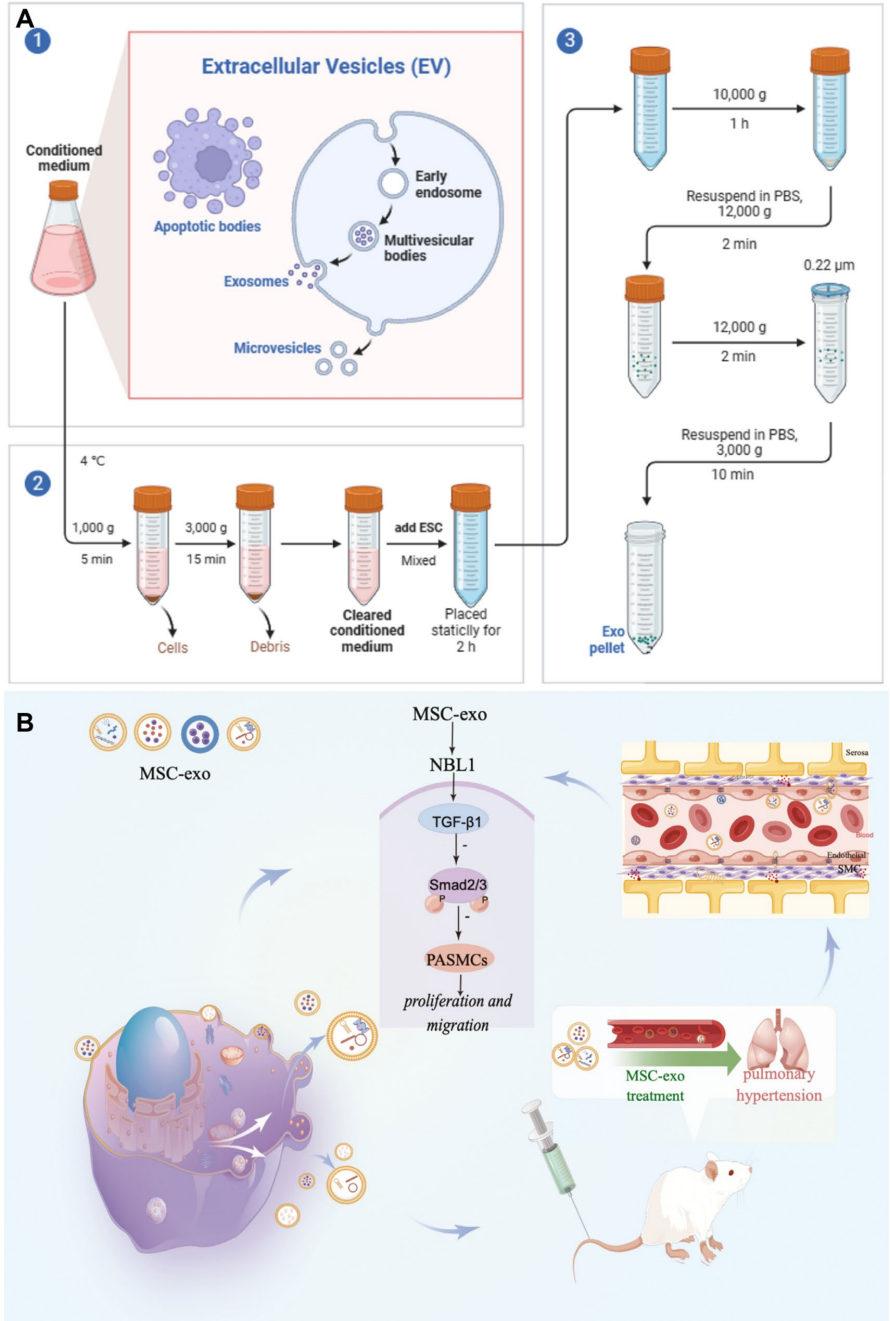
(**A**) Preparation of MSC-Exos targeting epidermal growth factor receptor/Erb-B2 receptor tyrosine kinase 2 heterodimerization for the treatment of hypoxic pulmonary hypertension. Reproduced with permission [130]. Copyright. 2025, Springer Nature. (**B**) Tibetan MSC-Exos induce downregulation of TGF-β1 to attenuated pulmonary vascular. Reproduced with permission [131]. Copyright. 2024, Oxford University Press

Therapeutic MSC-Exos have been extensively investigated in hypoxia-induced pulmonary hypertension models. MSC-Exos attenuate hypoxia-induced elevations in right ventricular systolic pressure and right ventricular hypertrophy while alleviating pulmonary vascular remodeling in rats. Mechanistically, these protective effects are mediated through modulation of multiple signaling pathways, including yes associated protein 1 /secreted phosphoprotein 1 (YAP1/SPP1), epidermal growth factor receptor/Erb-B2 receptor tyrosine kinase 2 heterodimerization-related pathways, and heat shock protein 90 alpha family class A member 1 (Hsp90aa1)/ERK/pERK pathway [128–130] (Fig. 5). Notably, MSC-Exos from Tibetan populations exhibit enhanced therapeutic efficacy. Tibetan MSC-Exos markedly attenuate pulmonary vascular remodeling and right ventricular hypertrophy in HAPH rats while suppressing PASMCs proliferation and migration. Transcriptomic analyses revealed upregulation of transforming growth factor-β (TGF-β) pathway related genes, including *Nbl1*,* Id2*,* Smad6*, and *Ltbp1*, suggesting altitude-adaptive molecular signatures may contribute to their protective effects [131] (Fig. 6).

Beyond MSC-Exos, integrin β1-modified telocyte-derived exosomes also show therapeutic potential. Overexpression of integrin β1 in telocytes elevated miR-429-3p enrichment in their exosomes without altering telocyte biological properties. These exosomes attenuated hypoxia-induced PASMCs proliferation, migration, and inflammation by directly targeting Rac1. In vivo administration alleviated pulmonary vascular remodeling, right ventricular systolic pressure, right ventricular hypertrophy, and mitigated inflammatory cytokines such as IL-6 and IL-1β in hypoxia-induced pulmonary hypertension mice [132].

**Fig. 7:**
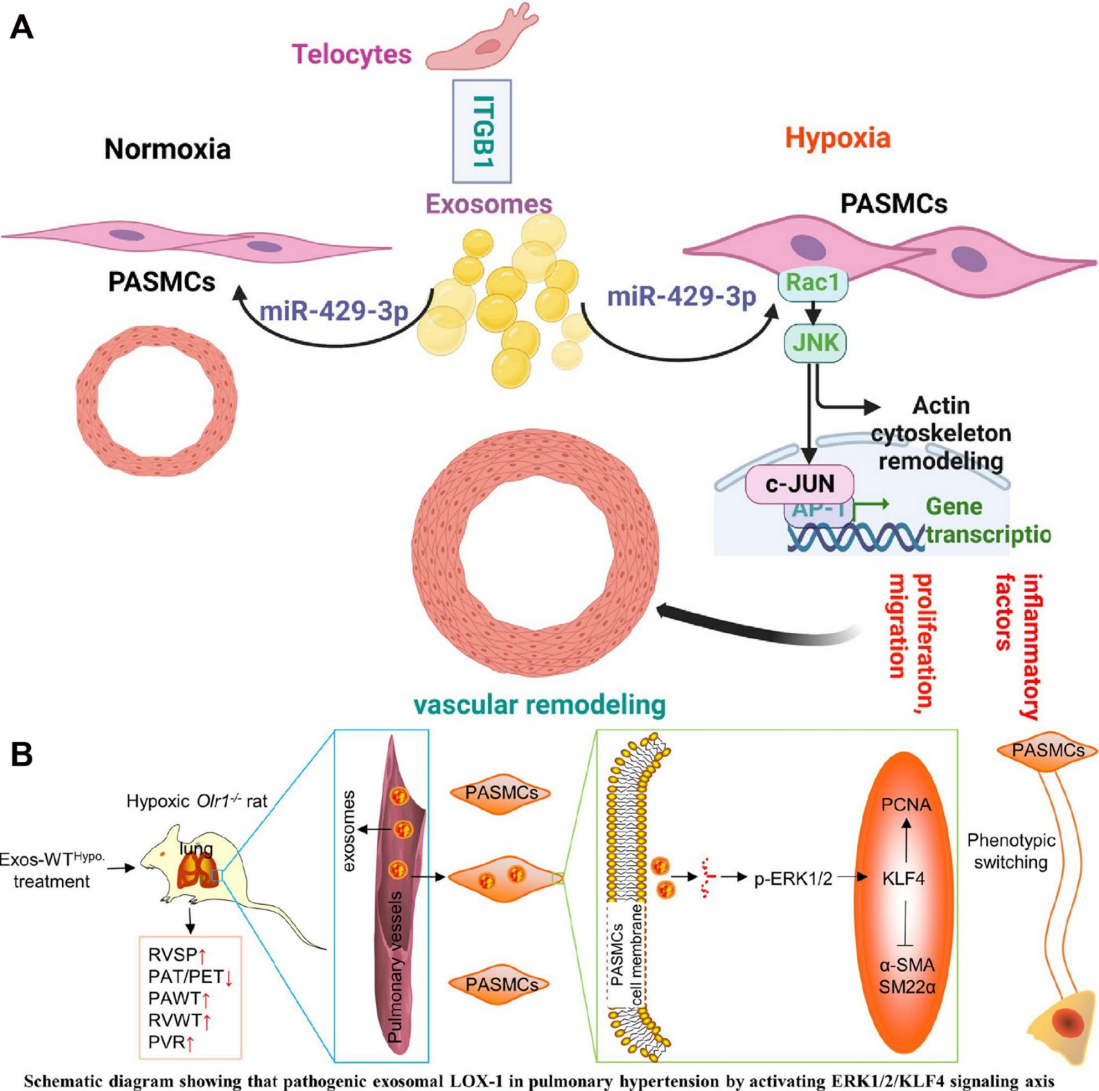
(**A**) Exosomes enriched by miR-429-3p derived from Integrin beta-1 modified telocytes alleviates hypoxia-induced pulmonary hypertension through regulating Rac1 expression. Reproduced with permission [132]. Copyright. 2024, Springer Nature. (**B**) Plasma-derived exosomes confer hypoxic pulmonary hypertension by delivering lectin like oxidized LOX-1 into PASMCs. Reproduced with permission [133]. Copyright. 2023, Elsevier

In contrast, accumulating evidence indicates that exosomes can also exert pathogenic effects in HAPH, depending on their cellular origin and cargo composition. Plasma-derived exosomes from hypoxia-induced pulmonary hypertension rats contain elevated lectin-like oxidized low-density lipoprotein receptor-1 (LOX-1), which is transferred to PASMCs and promotes phenotypic switching, proliferation, and migration through activation of the ERK1/2-Krüppel-like factor 4 signaling axis [133] (Fig. 7). Similarly, plasma exosomal miR-211 is upregulated in hypoxic pulmonary hypertension and correlates with disease severity, promoting PASMCs proliferation via negative regulation of Ca^2+^/calmodulin-dependent kinase1/peroxisome proliferator-activated receptors-γ axis pathway [134].

**Fig. 8:**
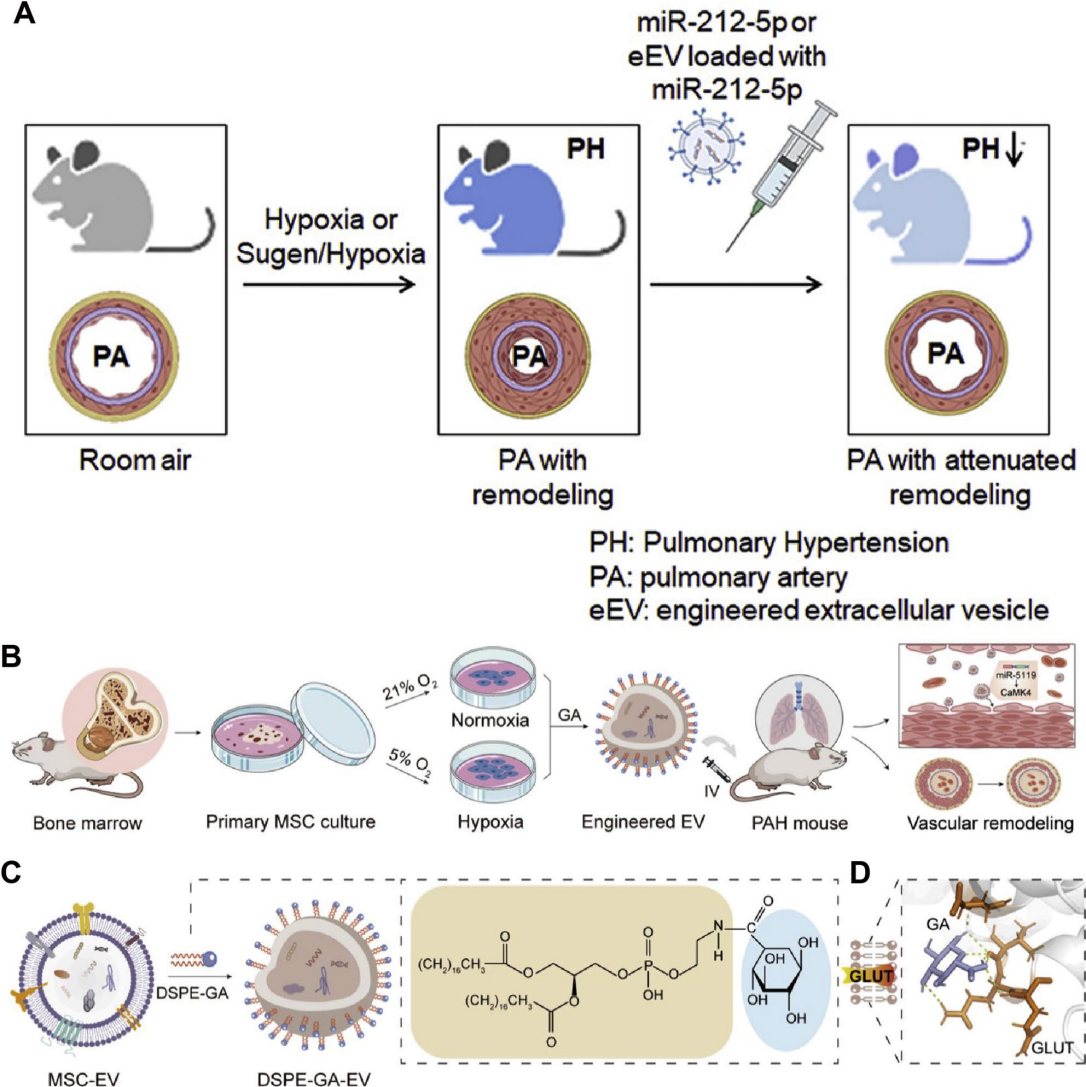
(**A**) Extracellular vesicles loaded with miR-212-5p attenuated hypoxia-induced pulmonary hypertension in mice. Reproduced with permission [136]. Copyright. 2022, Elsevier. (**B**) Applying engineered hypoxia-induced mesenchymal stromal cell-derived extracellular vesicles for pulmonary arterial hypertension, (**C**) Preparation of glucuronic acid-modified mesenchymal stromal cell-derived extracellular vesicles, (**D**) Simulation of glucuronic acid targeting to glucose transport-1 locating at the surface of PASMCs. Reproduced with permission [137]. Copyright. 2024, American Chemical Society

Hypoxia also enhances exosome secretion from PAECs. PAECs-derived exosomes enriched in 15-lipoxygenase 2 activate STAT3 signaling, promoting endothelial proliferation, migration, and angiogenic activity. Pharmacological blockade of exosome release mitigates pulmonary vascular remodeling and right ventricular dysfunction in hypoxic mice, highlighting the pathogenic contribution of endothelial exosomes under hypoxic stress [135]. Advances in exosome engineering further expand their therapeutic potential as natural nanocarriers. Endothelial extracellular vesicles enriched with the anti-proliferative miRNA miR-212-5p (212-eEVs) enable targeted pulmonary delivery following intratracheal administration and effectively attenuate hypoxia-induced pulmonary hypertension in mice [136]. Moreover, hypoxia-induced and glucuronic acid-modified mesenchymal stromal cell-derived extracellular vesicles exhibit enhanced targeting efficiency toward PASMCs and superior therapeutic efficacy compared with native vesicles. Mechanistically, miR-5119, associated with calcium signaling dysregulation in pulmonary hypertension, was identified as a key mediator of these effects [137] (Fig. 8). Exosomes, as a kind of natural nanomaterial, have unique therapeutic potential in HAPH. Exosomes from different sources are involved in cell communication, thereby improving pulmonary vascular remodeling and right ventricular load [128–137]. These findings highlight the dual role of exosomes in HAPH, functioning as both pathogenic mediators and therapeutic nanoplatforms depending on their cellular origin and molecular cargo. While native and engineered exosomes offer compelling advantages as natural nanomaterials, challenges related to cargo heterogeneity, large-scale production, targeting specificity, and long-term safety remain to be addressed. Future studies integrating exosomes engineering with macrophage- or vascular cell-specific targeting strategies may further enhance their translational potential for HAPH therapy.

### Organic nanocarriers

Beyond exosomes, organic nanocarriers have been actively explored for HAPH therapy due to their tunable physicochemical properties, including size, surface chemistry, and drug-loading capacity. Compared with natural nanomaterials, organic nanocarriers offer higher engineering flexibility but generally rely more heavily on synthetic targeting ligands and stimulus-responsive designs, which may introduce additional translational complexity [138].

**Fig. 9:**
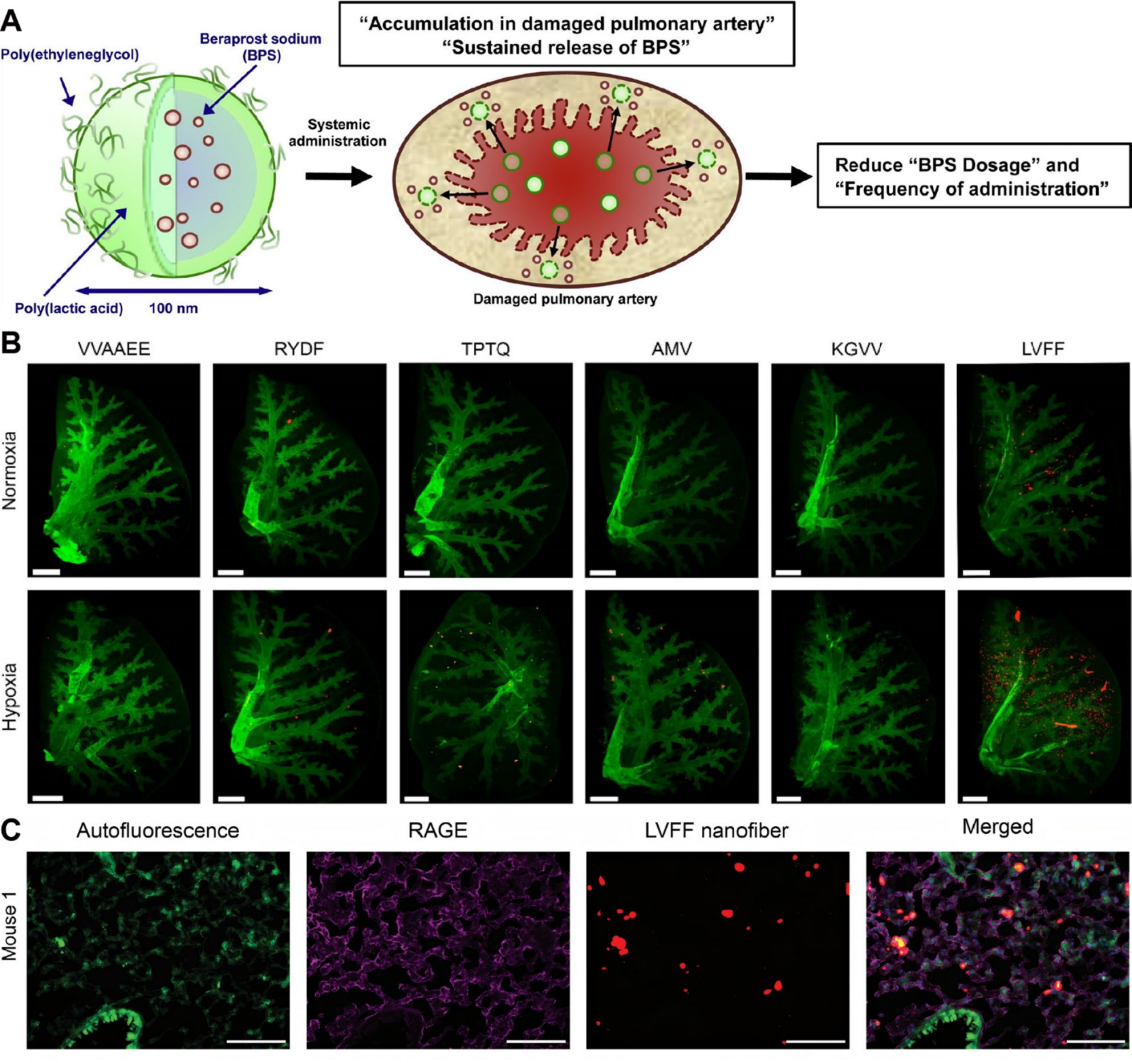
(**A**) BPS-NP, prepared from poly (lactic acid) and mPEG-PLA block copolymer, target hypoxia-injured pulmonary vasculature and provide sustained drug release, thereby attenuating pulmonary arterial remodeling and right ventricular hypertrophy. Reproduced with permission [142]. Copyright. 2015, Elsevier. (**B**) RAGE-targeted LVFF nanofiber localizes to lung with hypoxia-induced pulmonary hypertension (**C**) Peptide amphiphile nanofibers targeted to the RAGE accumulate within diseased lungs of mice with hypoxia-induced pulmonary hypertension following intravascular delivery. Reproduced with permission [139]. Copyright. 2021, Wiley

Self-assembled peptide amphiphile nanofibers have been developed that incorporate targeting epitopes to enhance lung specificity. Notably, peptide amphiphile nanofibers designed to recognize both the receptor for advanced glycation end-products (RAGE) and angiotensin-converting enzyme (ACE) I, via the targeting sequence LVFFAED, achieved selective accumulation in lung tissue in a chronic hypoxia-induced pulmonary hypertension mouse model [139] (Fig. 9). While this strategy establishes effective pulmonary targeting in vivo, mechanistic evidence linking nanofiber accumulation to specific vascular cell types remains limited, and therapeutic outcomes were primarily assessed at the tissue level rather than through cell-resolved analyses.

**Fig. 10:**
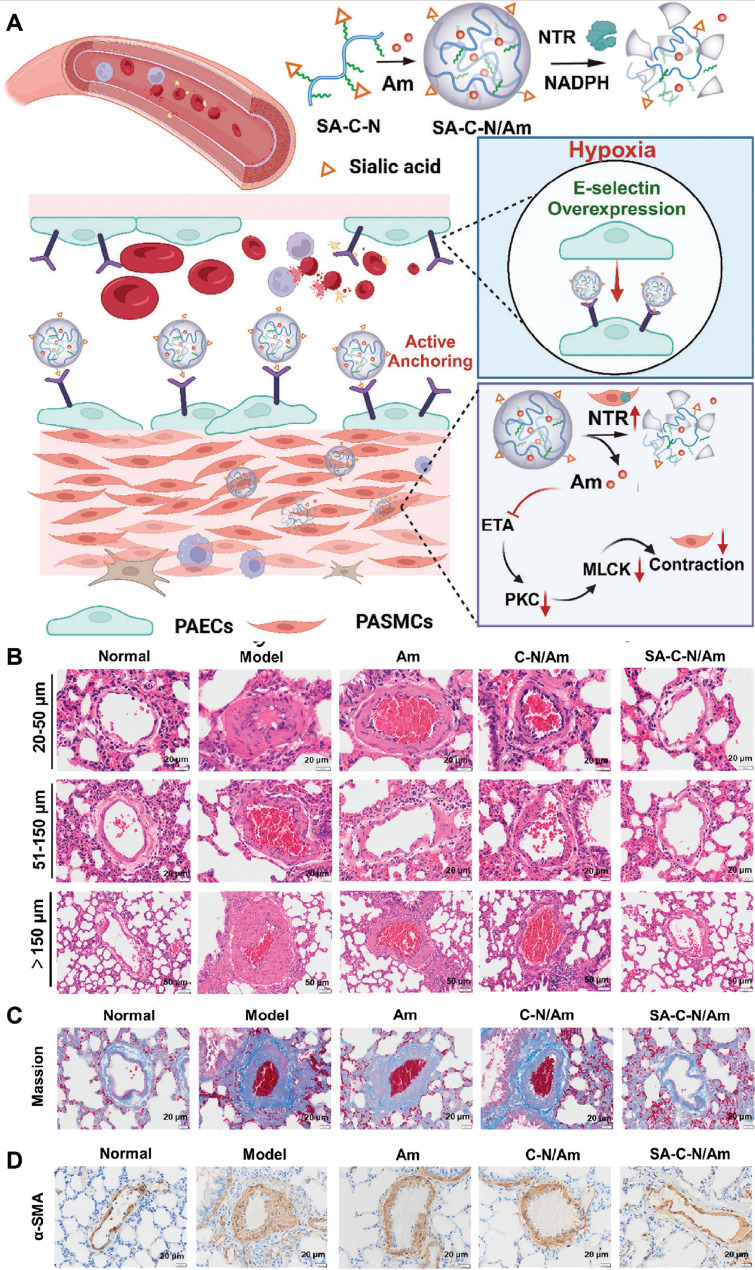
(**A**) Sialic acid-modified nanocarrier encapsulating ambrisentan targets E-selectin on PAECs, enabling efficient delivery to PASMCs and treating hypoxic pulmonary hypertension. (**B**) H&E staining results of pulmonary arterioles of different diameters in the left lower lobe of rat lung. (**C**) Masson staining results of pulmonary arterioles in the left lower lobe of rat lung. (**D**) α-smooth muscle actin immunohistochemical results of pulmonary arterioles in the left lower lobe of rat lung. Reproduced with permission [140]. Copyright. 2024, Wiley

**Fig. 11:**
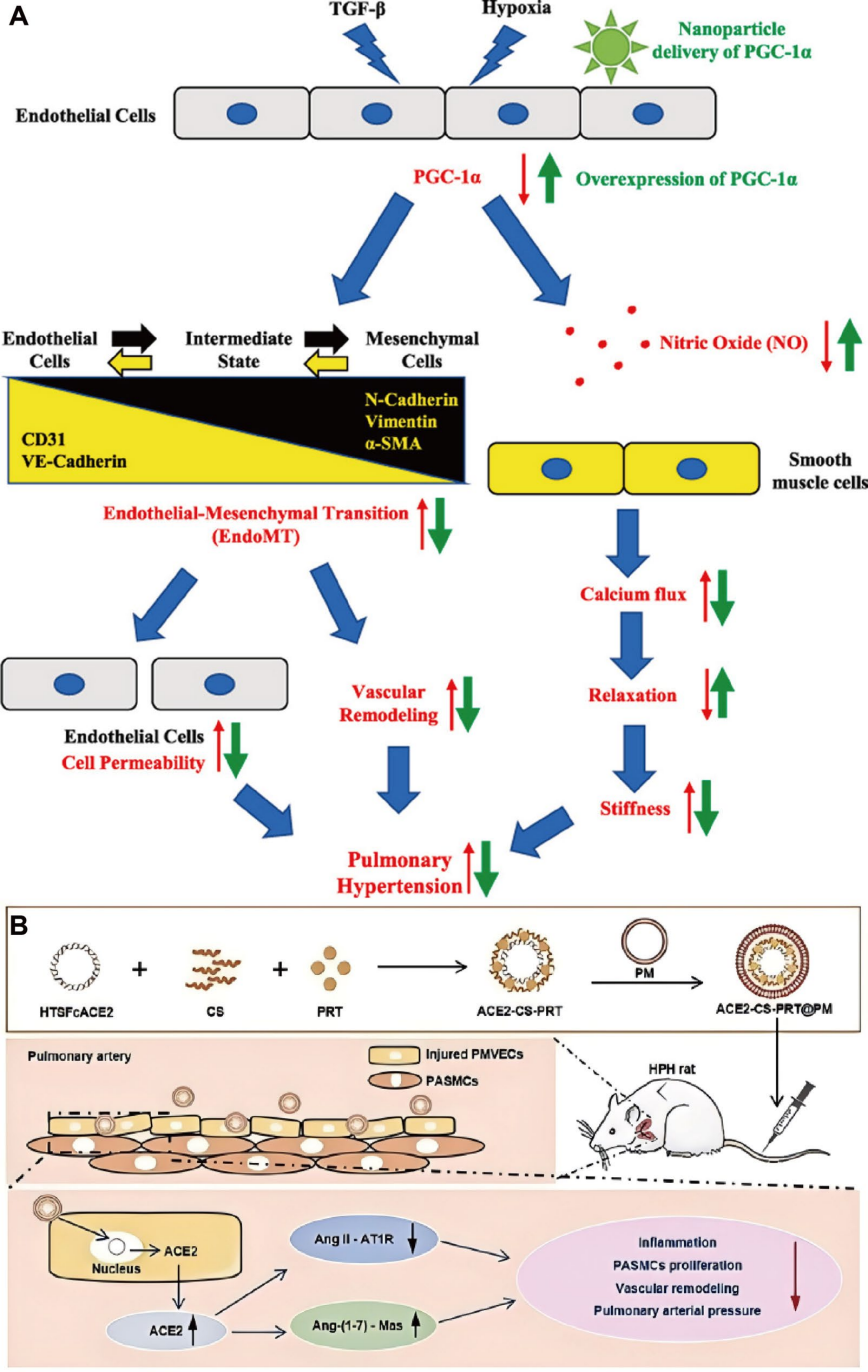
(**A**) Nanoparticle endothelial delivery of PGC-1α attenuates hypoxia-induced pulmonary hypertension by attenuating endothelial-to-mesenchymal transition-caused vascular wall remodeling. Reproduced with permission [144]. Copyright. 2022, Elsevier. (**B**) Biomimetic nanoparticle-mediated target delivery of hypoxia-responsive plasmid of ACE2 to reverse hypoxic pulmonary hypertension. Reproduced with permission [145]. Copyright. 2023, American Chemical Society

Endothelial adhesion molecules represent attractive targets for selective drug delivery in HAPH. E-selectin is upregulated in PAECs under hypoxic conditions and contributes to disease progression. To exploit this feature, a sialic acid-modified nanoparticle system encapsulating ambrisentan was developed to specifically target E-selectin-expressing PAECs. Incorporation of hypoxia-responsive nitrobenzene moieties enabled drug release in reductive microenvironments characterized by elevated nitroreductase activity. This nanoplatform enhanced endothelial uptake, facilitated intercellular transfer to PASMCs, and significantly lowered pulmonary arterial pressure and right ventricular hypertrophy in vivo, lending support to both targeting precision and therapeutic efficacy [140] (Fig. 10). Nanoparticle-based formulations have also been employed to optimize the delivery of clinically approved vasodilators. Prostaglandin I2 can be secreted by PAECs, where it exerts vasodilatory effects and restrains PASMCs activity [141]. Its analogue, beraprost sodium, has also shown therapeutic benefits in pulmonary hypertension. To enhance the targeting efficiency of beraprost sodium and minimize its side effects, beraprost sodium-loaded nanoparticles (termed BPS-NP) were successfully developed. In vitro, BPS-NP showed sustained-release characteristics and prolonged circulation time. In vivo, BPS-NP preferentially accumulated in the peripheral pulmonary arteries of injured lungs, and a once-weekly intravenous injection of BPS-NP (20 µg/kg) ameliorated hypoxia-induced pulmonary vascular remodeling and right ventricular hypertrophy [142]. Although these results support enhanced pharmacokinetics and pulmonary targeting in vivo, the long-term safety and off-target accumulation of repeated nanoparticle administration were not comprehensively evaluated, limiting conclusions regarding chronic HAPH treatment. In addition, imatinib mesylate, a PDGF receptor tyrosine kinase inhibitor, has been shown to ameliorate pulmonary hypertension by reversing pulmonary vascular remodeling. To achieve precise pulmonary delivery and sustained drug release, imatinib mesylate-loaded liposomes (termed IM-LPs) were developed. IM-LPs, prepared via the transmembrane gradient method, had an average size of 101.6 ± 50.80 nm, a zeta potential of 19.66 ± 0.55 mV, a polydispersity index of 0.250, and an entrapment efficiency of 81.96%±0.98%, showing a sustained release profile. Compared with plain imatinib mesylate solution, IM-LPs exhibited stronger targeting ability to PASMCs, as evidenced by enhanced intracellular accumulation of Rhodamine B in PASMCs observed through fluorescence microscopy. In vivo pharmacokinetic studies in rats revealed that intratracheal administration of IM-LPs prolonged the half-life and retention time of imatinib mesylate compared with intratracheal and intravenous administration of plain imatinib mesylate solution. Additionally, IM-LPs showed no cytotoxicity to PASMCs in vitro. These results indicate that IM-LPs have superior effects in pulmonary hypertension treatment, with their sustained release property and enhanced targeting to PASMCs being key contributors, making them a promising formulation for pulmonary delivery of imatinib mesylate. However, before considering clinical application, further in vivo distribution, safety analysis and translational research are indispensable [143]. Endothelial-to-mesenchymal transition has been recognized as a major contributor to endothelial dysfunction in pulmonary hypertension. In both pulmonary hypertension patients and hypoxia-induced pulmonary hypertension mouse models, PAECs exhibit downregulated expression of peroxisome proliferator-activated receptor gamma coactivator 1-alpha (PGC-1α). To address this, nanoparticles delivering PGC-1α have been designed to specifically target PAECs, thereby blocking endothelial-to-mesenchymal transition and reversing pulmonary hypertension progression. Overexpression of PGC-1α also restores eNOS expression in PAECs of hypoxia-exposed mouse lung tissue. Moreover, co-culture studies of PAECs and PASMCs confirm that PGC-1α overexpression in PAECs increases nitric oxide release, which may diffuse to PASMCs, activate specific protein kinases, and limit calcium influx, ultimately inducing PASMCs relaxation and attenuating hypoxia-induced pulmonary arterial stiffening [144]. ACE2-CS-PRT@PM system, a nanoparticle platform specifically designed to target PAECs. In this strategy, a hypoxia-responsive plasmid encoding ACE2 was constructed using an endothelial-specific Tie2 promoter and a hypoxia response element, followed by the development of a biomimetic nanoparticle delivery system. This system enables targeted delivery to injured PAECs, where it induces encoding ACE2 overexpression under hypoxic conditions. In hypoxia-induced pulmonary hypertension rats, ACE2-CS-PRT@PM effectively counteracted PASMCs proliferation, attenuated pulmonary vascular remodeling, restored the balance of the pulmonary renin-angiotensin system, and improved the inflammatory microenvironment. These effects collectively ameliorated hemodynamic dysfunction and vascular abnormalities, largely reversing pulmonary hypertension progression, with no detectable toxicity. Such findings highlight its promising therapeutic potential [145] (Fig. 11). Collectively, organic nanocarrier-based interventions in HAPH predominantly target endothelial dysfunction and PASMCs hyperproliferation, with most evidence derived from rodent models. While in vivo efficacy is consistently observed, cross-study comparison is hindered by heterogeneity in administration routes, targeting strategies, and outcome measures. Moreover, few studies directly compare organic nanocarriers with natural vesicles or conventional therapies, making it difficult to define their relative advantages.

### Gas-delivery nanomedicine

Gas therapy has emerged as a promising clinical strategy for a variety of diseases. The integration of nanotechnology with gas therapeutics has given rise to nanoscale gas delivery systems, which not only enhance the stability and bioavailability of therapeutic gases but also enable controlled and targeted release [146]. Within the respiratory field, the combination of inhaled gases and nanocarriers holds particular therapeutic potential, offering a novel avenue for the treatment of pulmonary disorders.

Given the critical role of nitric oxide in HAPH, early studies have attempted to target the nitric oxide pathway using nanomedicine. These formulations, based on hydrogel-like polymer composites encapsulating nitric oxide nanoparticles, were able to sustain nitric oxide release for up to 8 h without affecting endothelial cell viability or inducing inflammatory responses. In hypoxic mice, nitric oxide nanoparticles effectively targeted the pulmonary vasculature to induce vasodilation. Compared with free nitric oxide, this strategy overcomes the extremely short half-life of nitric oxide and enhances its targeted delivery [147]. Beyond gas delivery, nanotechnology has also been leveraged to modulate hypoxia-activated intracellular signaling pathways. Hybrid nanoparticles composed of low-molecular- weight polyethylenimine combined with pH-responsive cyclodextrin derivatives or poly (lactic-co-glycolic acid) were developed for siRNA-mediated mTOR silencing. These systems effectively restricted PASMCs proliferation and promoted apoptosis in vitro, and attenuated pulmonary vascular remodeling in hypoxic animal models [148]. While these findings support mTOR as a viable therapeutic target, the delivery efficiency, cell-type specificity, and long-term safety of such cationic polymer-based systems remain key challenges for clinical translation. Collectively, current gas-related nanotherapeutic strategies for HAPH primarily exert their benefits through vasodilation and suppression of PASMCs hyperproliferation. Yet, their limited engagement with immune and inflammatory mechanisms suggests that future designs may benefit from integrating gas delivery with immunomodulatory nanoplatforms, thereby achieving more comprehensive regulation of hypoxia-driven vascular remodeling.

### Metal-based nanocarriers and metal-organic frameworks

Metal-based nanostructures possess tunable size, shape, and photophysical properties, enabling applications in bioimaging and photoresponsive therapy[149]. These features enable them to scavenge reactive species, regulate pathological signaling, and act as drug carriers, making them promising multifunctional platforms for pulmonary hypertension therapy [150]. Their therapeutic relevance in HAPH varies substantially depending on metal composition, administration route, and targeted cell populations.

Metal-organic skeletons are a drug delivery method, which have good biocompatibility and strong drug delivery capacity in vivo. Curcumin derivative WZ35 has better biological safety than the curcumin. By preparing a copper-based metal organic framework to package WZ35, a nano-drug delivery system named MOFCu@WZ35 nanoparticles was formed. This system can target PASMCs and restrain hypoxia-induced PASMCs hyperproliferation, showing potential for the treatment of HAPH, but in vivo pharmacokinetic and toxicity evaluations were not conducted in the same study, underscoring the need for further investigation into long-term safety and off-target effects before clinical application can be considered [151].

**Fig. 12:**
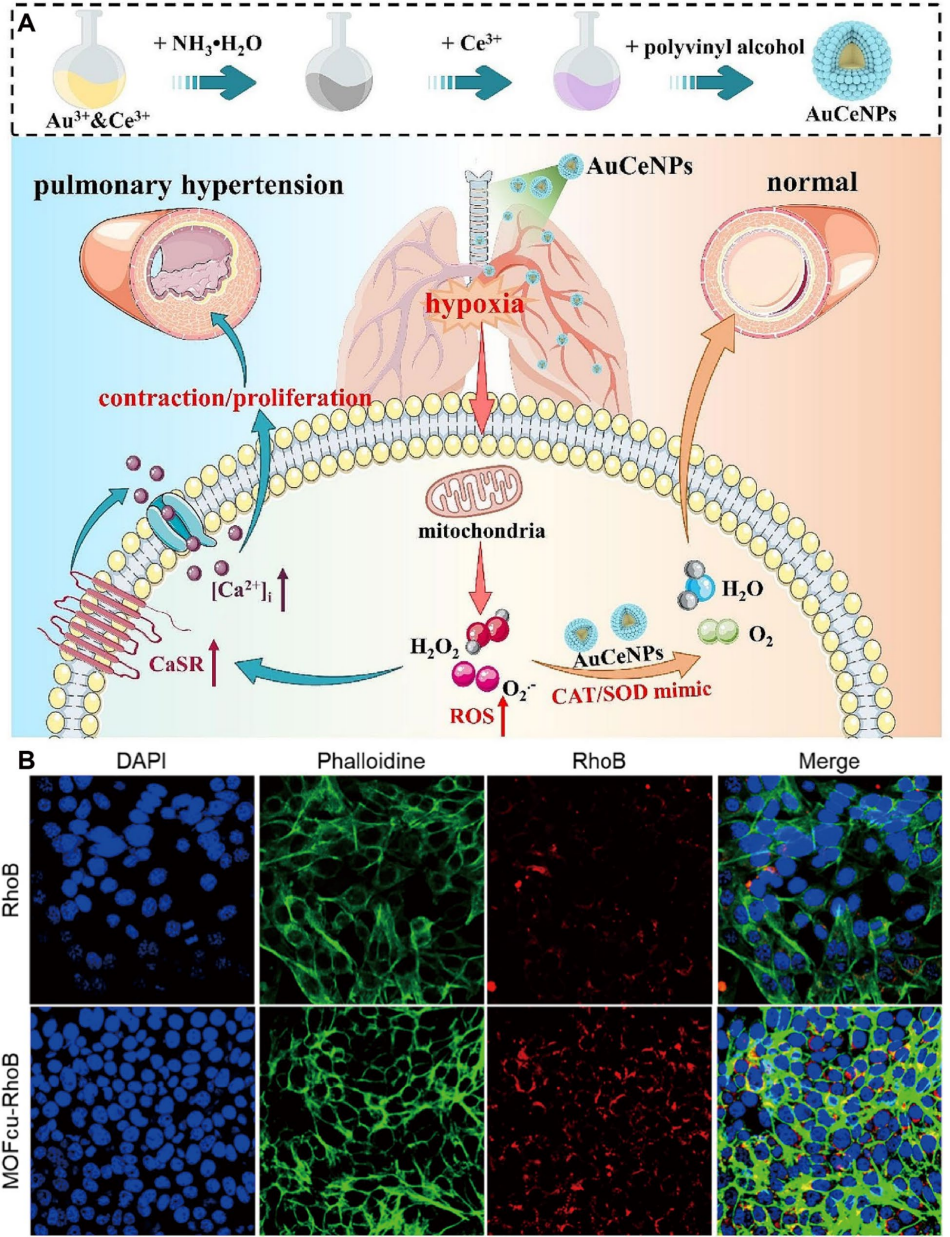
(**A**) Au-modified ceria nanozyme can effectively prevent and treat hypoxia-induced pulmonary hypertension by scavenging ROS. Reproduced with permission [152]. Copyright. 2024, BMC. (**B**) The cellular uptake of MOFCu-Rhomine B and Rhomine B detected by the laser scanning confocal microscope. Reproduced with permission [151]. Copyright. 2023, Wiley

Which exhibit catalase- and superoxide dismutase-like activities, have been widely explored for reactive oxygen species scavenging. AuCeNPs, consisting of a gold nanocore and ceria shell, were shown to reach PASMCs following nebulized inhalation and modulate the activity and expression of extracellular calcium-sensing receptors in hypoxic rats, thereby attenuating pulmonary hypertension [152]. Thus, it can prevent and treat hypoxia-induced pulmonary hypertension. Moreover, nebulized AuCeNPs have no effect on systemic arterial pressure, liver and kidney function, plasma Ca^2+^ level and blood biochemical parameters of rats, these data suggest acceptable short-term safety, but long-term in vivo toxicity and distribution remain to be clarified [152] (Fig. 12). Encapsulating existing therapeutic drugs can improve targeting. Therefore, a nanosystem named Sil@nanoMIL-89 was prepared by encapsulating sildenafil using the highly porous iron-based metal-organic framework, which released sildenafil within 6 h. It will be continuously released within 72 h. At concentrations as high as 100 µg/ml, Sil@nanoMIL-89 shows no significant toxicity to human blood-derived endothelial cells and achieves vasodilation function. Given the drug’s continuous release ability, further evaluation of its ability in HAPH animal models is needed [153]. Metal-based nanostructures offer multifunctional advantages for HAPH therapy, including oxidative stress mitigation, enhanced drug delivery, and sustained pharmacological effects. Nevertheless, current evidence is predominantly derived from cellular and short-term animal studies, with limited mechanistic integration across vascular, immune, and inflammatory pathways. Addressing long-term safety, biodistribution, and cell-type specificity will be essential for advancing these platforms toward translational application.

## Nanoplatform-enabled macrophage immunomodulation in HAPH

### Nanoplatform-enabled modulation of pulmonary macrophage phenotypes and functions

Recent advances in nanotechnology have enabled increasingly precise manipulation of pulmonary macrophages, allowing selective regulation of their recruitment, polarization, inflammatory signaling, and metabolic programs within the lung microenvironment. These nanoplatforms function not merely as passive drug carriers but as active immunomodulatory systems capable of reshaping macrophage behavior through defined molecular and cellular mechanisms.

One major strategy involves interfering with macrophage recruitment by targeting chemokine and adhesion pathways. For instance, lipid nanoparticle-mediated delivery of CCR2 siRNA effectively suppressed CCR2 expression in pulmonary macrophages, leading to attenuated accumulation of monocyte-derived macrophages in vivo. While short-term administration demonstrated favorable biocompatibility both in vitro and in vivo, long-term immune consequences and effects on systemic monocyte trafficking remain insufficiently explored [154]. Similarly, platelet membrane-coated nanoparticles exploit platelet-inflamed endothelium interactions to achieve preferential accumulation at pulmonary inflammatory sites. This biomimetic approach limited macrophage infiltration in murine models and showed no overt histopathological damage to major organs, supporting acceptable short-term biosafety, however, its dependence on disease-specific endothelial activation may limit generalizability across inflammatory contexts [155]. Beyond recruitment, several nanoplatforms directly modulate macrophage inflammatory phenotypes. In a monocrotaline-induced pulmonary arterial hypertension rat model, intratracheal delivery of pitavastatin-loaded nanoparticles enabled prolonged retention in AMs and small pulmonary arteries, resulting in more effective suppression of vascular inflammation and remodeling than free or systemically administered pitavastatin. Delayed administration after disease establishment induced partial regression of pulmonary arterial hypertension and improved survival, highlighting the therapeutic advantage of lung-targeted nanoplatforms that engage macrophage-rich microenvironments [156]. Building on this macrophage-centric concept, inflammatory transcriptional regulation has emerged as a key targetable axis in pulmonary arterial hypertension. Elevated NF-κB activity was observed in both small pulmonary arterial lesions and AMs in lungs from pulmonary arterial hypertension patients, underscoring the close linkage between macrophage-driven inflammation and vascular remodeling. In monocrotaline-induced pulmonary arterial hypertension rats, intratracheal delivery of polymeric nanoparticles enabled sustained pulmonary retention and efficient delivery of an NF-κB decoy oligonucleotide. This nanoparticle-mediated blockade of NF-κB signaling markedly suppressed macrophage-associated inflammatory responses and vascular cell proliferation, thereby attenuating pulmonary arterial remodeling and pulmonary arterial hypertension progression. Therapeutic intervention initiated after disease establishment also improved survival, reinforcing the translational relevance of inhalable nanoplatforms that directly modulate macrophage-centered inflammatory signaling in pulmonary arterial hypertension [157]. Beyond drug- or gene-loaded nanocarriers, certain nanomaterials exhibit intrinsic immunomodulatory properties relevant to pulmonary arterial hypertension. Iron-based metal-organic frameworks, such as MIL-89 and its PEGylated derivative, accumulate efficiently in the lung and directly modulate macrophage inflammatory activity. In macrophages, MIL-89-based nanoparticles suppress iNOS activity, indicating a direct regulatory effect on macrophage-driven inflammation, while demonstrating favorable pulmonary retention and tolerability in vivo [158]. Fe-capsaicin nanozymes suppressed NF-κB activation in macrophages in vitro and attenuated pulmonary IL-6 and iNOS expression in lipopolysaccharide-challenged mice, while exhibiting minimal organ toxicity, supporting nanozyme-based redox regulation as a viable immunomodulatory strategy [159]. Fluorous-tagged peptide nanoparticles demonstrated consistent macrophage internalization in vitro and anti-inflammatory efficacy in vivo, providing coherent cross-model validation of macrophage targeting [160]. Macrophage-directed gene silencing represents a more mechanistically precise strategy. Gan Zhao et al. developed macrophage-targeted lipid nanoparticles encapsulating TGF-β-activated kinase 1 siRNA, which suppressed pro-inflammatory signaling in macrophages and alleviated influenza-induced lung injury. This study incorporated evaluations of cytotoxicity, immunogenicity, and off-target risk, thereby strengthening its translational relevance [184]. Phosphatidylserine-decorated platforms similarly exploit endogenous “eat-me” signals to enhance macrophage uptake. Dexamethasone-loaded phosphatidylserine-decorated poly (lactic-co-glycolic acid) nanoparticles reduced pulmonary inflammation and acute lung injury in vivo, although the authors appropriately raised concerns regarding potential coagulation perturbations at higher exposure levels, underscoring the dose-dependent safety considerations unique to macrophage-targeting nanomaterials [161]. In addition to inflammatory signaling, macrophage polarization and function are tightly coupled to intracellular redox and metabolic states. Bone marrow mesenchymal stem cell-derived extracellular vesicles have been shown to function as efficient carriers of regulatory microRNAs, including miR-200b, which is markedly downregulated in lung tissues, PASMCs, and macrophages in monocrotaline-induced pulmonary arterial hypertension models. Mechanistically, bone marrow mesenchymal stem cell-derived extracellular vesicles transfer miR-200b into macrophages, promoting their polarization toward an anti-inflammatory M2 phenotype and thereby alleviating pulmonary arterial hypertension-associated pathological features in vivo [185]. Adipose-derived stem cell exosomes attenuated lipopolysaccharide-induced macrophage hyperactivation, reducing macrophage accumulation and M1 polarization in bronchoalveolar lavage fluid of septic mice, accompanied by decreased pulmonary NOD-like receptor thermal protein domain associated protein 3 expression. Although these findings highlight potent immunomodulatory capacity in vivo, the absence of systematic biosafety and biodistribution analyses constrains translational interpretation [162]. Hyaluronic acid-modified flavonoid nanoparticles selectively targeted M1 AMs and restored mitochondrial function, thereby promoting M1-to-M2 repolarization and alleviating acute lung injury following nebulized delivery. These findings highlight the added therapeutic value of coupling phenotype modulation with mitochondrial repair [163]. Combination strategies further enhance therapeutic efficacy by integrating barrier penetration and immune regulation. Inhalable lipid nanoparticles co-encapsulating curcumin and N-acetylcysteine leveraged mucolytic activity to improve pulmonary delivery, resulting in lower oxidative burden, suppression of pro-inflammatory cytokines, and attenuation of macrophage and neutrophil accumulation in vivo. While effective in acute models, their durability under chronic inflammatory conditions remains unclear [164]. Extracellular vesicle-based platforms introduce an additional layer of regulatory complexity through microRNA-mediated signaling. Umbilical cord mesenchymal stem cell-derived small extracellular vesicles mitigated bleomycin-induced pulmonary fibrosis by modulating macrophage function, potentially via miR-146a-5p-mediated suppression of M1 polarization [165]. Likewise, exosomal miR-124-3p promoted macrophage repolarization toward an anti-inflammatory phenotype in lung transplantation-associated acute lung injury, with consistent in vitro and in vivo evidence linking NF-κB inhibition to improved lung repair and function [166]. Nevertheless, variability in vesicle composition and challenges in scalable production remain unresolved barriers to clinical translation. More advanced biomimetic systems actively transfer immunoregulatory functions. ROS-responsive nanoparticles cloaked with M2 macrophage membranes exploited natural homing properties and cytokine receptor-mediated neutralization to suppress inflammatory signaling in vivo, effectively conferring anti-inflammatory functionality to diseased tissues [167]. Macrophage function is intimately coupled with their metabolic state, encompassing glycolysis, oxidative phosphorylation, and lipid metabolism. Pathological lactate accumulation has recently been recognized as a key metabolic driver of fibroblast activation and M2 macrophage polarization in idiopathic pulmonary fibrosis. A phosphatidylserine-conjugated PEGylated hollow mesoporous CeO₂ nanoplatform (CeO₂@PLP), co-load activity, and antifibrotic drug delivery, this system effectively suppressed lactate-driven M2 macrophage polarization and fibroblast differentiation. In bleomycin-induced pulmonary fibrosis models, CeO₂@PLP markedly improved lung function and attenuated extracellular matrix deposition, while exhibiting favorable in vivo safety, highlighting metabolic reprogramming of macrophages as a promising nanotherapeutic strategy for fibrotic lung diseases [168]. Finally, mitochondrial dysfunction represents a convergent node linking macrophage metabolism and inflammatory activation. A biomimetic apoptotic body-inspired nanoplatform (CeCPT@PSL) achieved preferential macrophage uptake via phosphatidylserine-mediated recognition while restoring mitochondrial redox homeostasis through encapsulated nanozyme complexes. In murine acute lung injury models, inhaled CeCPT@PSL concurrently dampened inflammatory cascades and repaired mitochondrial dysfunction, resulting in functional recovery of pulmonary physiology [169]. Complementing this approach, a self-assembled metabolic regulator that simultaneously inhibits glycolysis and the stimulator of interferon genes signaling pathway reprogrammed macrophage polarization, attenuated cytokine storm, and limited inflammatory macrophage infiltration in septic models, underscoring metabolic immunomodulation as a unifying strategy across inflammatory lung pathologies [170]. These studies demonstrate that nanoplatform-based strategies enable multifaceted regulation of pulmonary macrophages across multiple biological layers, including cellular recruitment, inflammatory signaling, polarization states, and metabolic reprogramming. By integrating precise cellular targeting with functional immunomodulation, such nanotherapeutic systems provide a powerful means to reshape macrophage-driven pathological cascades in pulmonary diseases. Importantly, several macrophage-targeted nanotherapeutic approaches have already been validated in experimental pulmonary arterial hypertension models, providing disease-specific proof-of-concept for this strategy. Cross-study comparisons reveal several persistent challenges, such as insufficient long-term biosafety evaluation, heterogeneity in experimental validation across in vitro and in vivo models, and limited assessment of translational scalability and clinical feasibility. Addressing these issues will be critical for accelerating the clinical translation of macrophage-targeted nanomedicines. Despite these limitations, the accumulated evidence highlights nanoplatforms as a highly promising and versatile therapeutic paradigm for macrophage-centered intervention. In particular, these advances offer valuable conceptual and technical insights for the development of macrophage-targeted nanotherapies in hypoxia-related pulmonary vascular diseases, including HAPH, where dysregulated macrophage activation and inflammation play pivotal pathogenic roles.

### Nanoplatform-enabled macrophage-targeted immunomodulation in HAPH

Nanomaterials have opened up unprecedented opportunities for the precise intervention of HAPH. By optimizing particle size, surface chemistry, and stimulus-responsive properties, nanocarriers can not only enhance drug accumulation and retention within the lung but also enable controlled release, combinatorial drug delivery, and real-time imaging [123, 171], thereby offering a promising approach for the long-term management of this progressive disease. Nevertheless, despite their ability to improve the pharmacokinetic profiles of therapeutics, the ultimate therapeutic benefit of nanotechnology still hinges on whether it can effectively modulate the key pathogenic drivers of HAPH. Among the complex pathological networks of HAPH, immune dysregulation, particularly macrophage-driven inflammation and vascular remodeling, emerges as a central mechanism. Increasing evidence indicates that macrophages act not only as effectors of inflammation but also as critical regulators and amplifiers of disease progression. Thus, leveraging nanotechnology to selectively reprogram macrophage recruitment, polarization, and metabolic states holds great potential to disrupt the vicious cycle of inflammation and vascular remodeling, thereby achieving genuine disease-modifying effects. This integrated perspective provides a conceptual framework for developing next-generation precision immunotherapies for HAPH.

Strategies that precisely target the recruitment, activation and phenotypic regulation of macrophages have received increasing attention as promising therapeutic approaches. In recent years, advancements in nanotechnology and bioengineered delivery systems have made the development of macrophage interventions possible, ranging from drug-loaded nanocarriers to naturally derived vesicles. These methods not only enhance the bioavailability of drugs within the lung system and reduce adverse drug reactions, but also offer opportunities for the selective regulation of macrophage-driven pathogenic processes (Table 3).

**Fig. 13:**
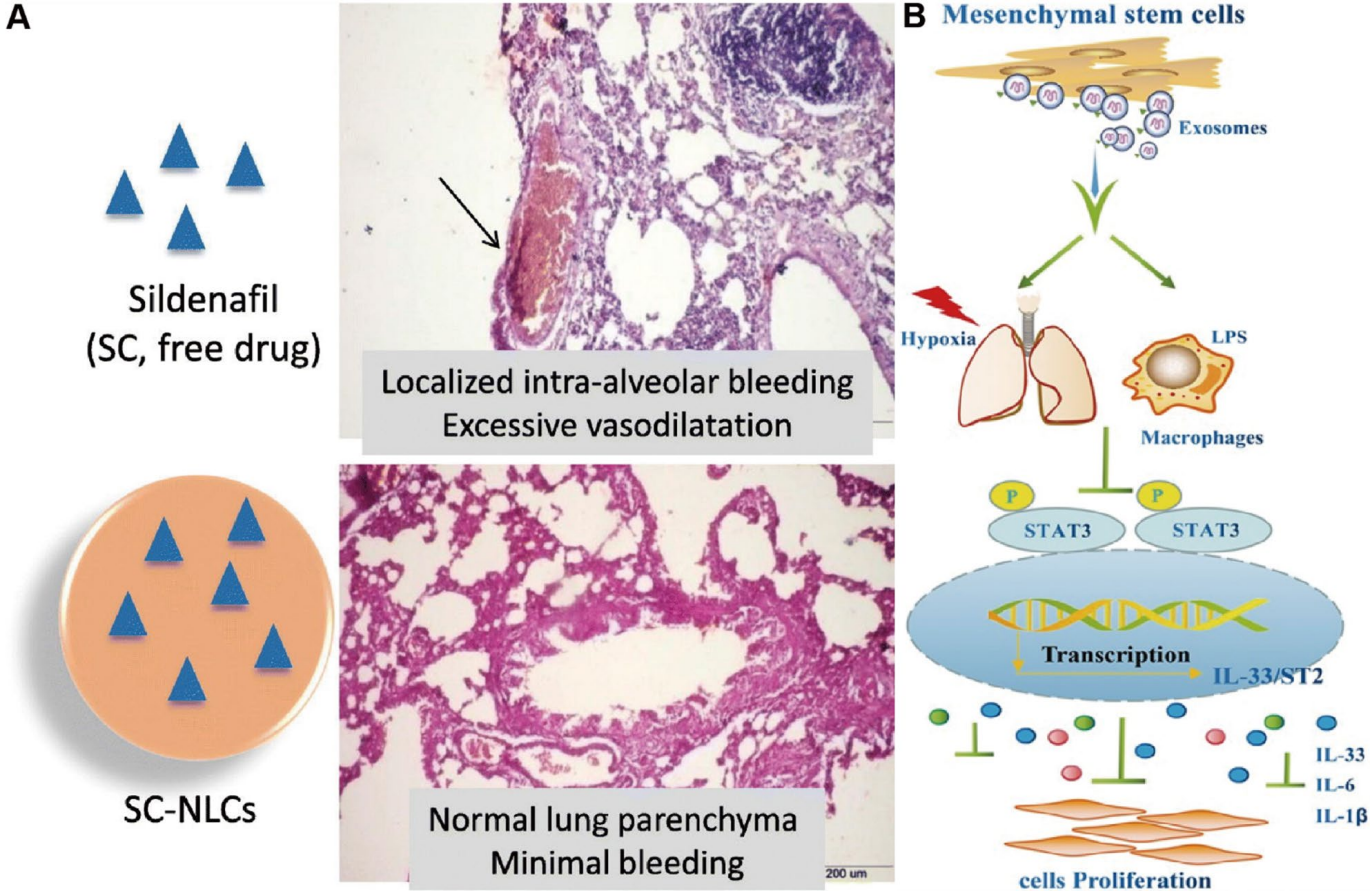
(**A**) Sildenafil-nanostructured lipid carriers, compared with free sildenafil, can reduce local alveolar haemorrhage. Reproduced with permission [172]. Copyright. 2018, Elsevier. (**B**) Human umbilical cord MSC-Exos promote macrophage M2 polarization while suppressing the IL-33/ST2 pathway. Reproduced with permission [177]. Copyright. 2023, Oxford University Press

Although sildenafil exhibits vasodilating action and can target macrophages, its non-selective vasodilatory effects, short half-life, and extensive first-pass metabolism limit its application for local pulmonary delivery. To address these limitations, nanostructured lipid carriers encapsulating sildenafil was developed. These nanostructured lipid carriers were formulated with solid lipids (precirol, stearic acid, or beeswax) in combination with oleic acid as the liquid lipid, and stabilized using PVA or poloxamer as emulsifiers. The system exhibited favorable colloidal and encapsulation stability for up to three months. In rat models, inhaled nanostructured lipid carriers-encapsulated sildenafil, compared with free sildenafil administration, attenuated local alveolar haemorrhage and led only to mild aggregation of particle-loaded AMs, accompanied by macrophage vacuolisation. This macrophage aggregation may underlie potential macrophage-targeting effects, although further validation in disease models is required [172] (Fig. 13).

**Fig. 14:**
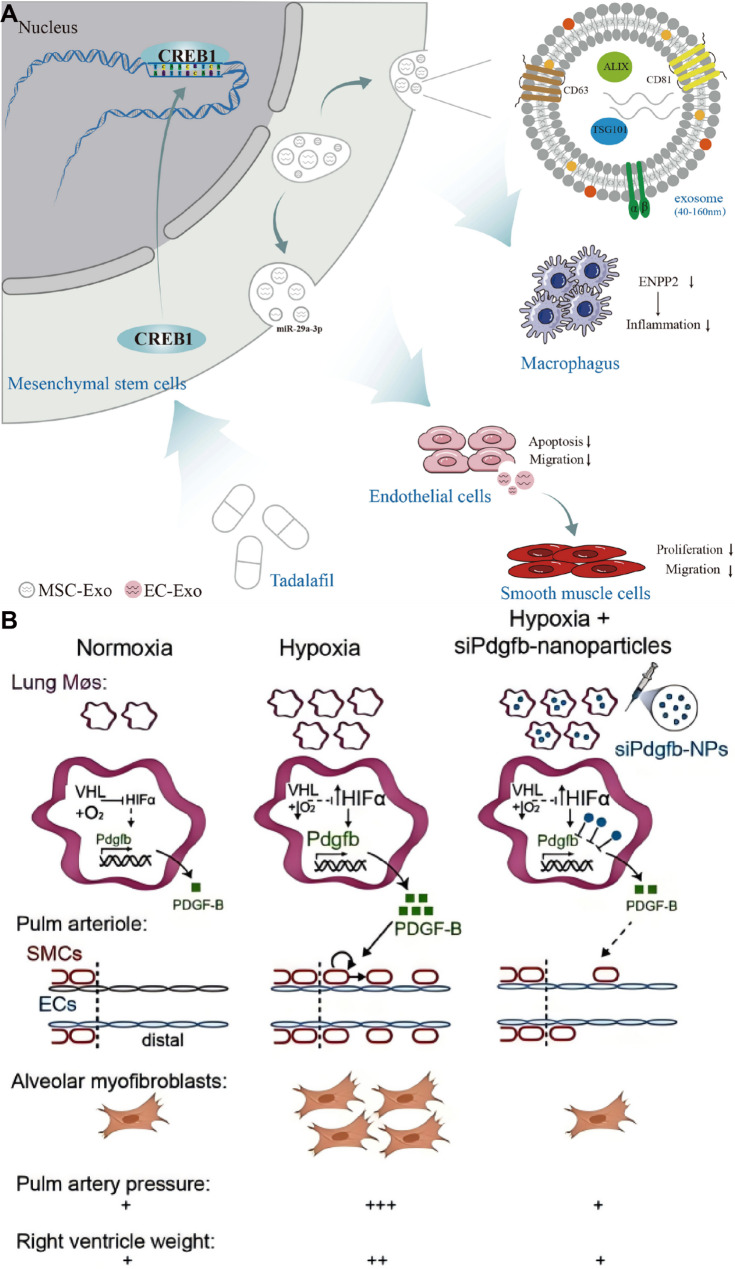
(**A**) Tadalafil enhances the therapeutic efficacy of MSC-Exos in pulmonary hypertension by upregulating miR-29a-3p. Reproduced with permission [175]. Copyright. 2024, Dove Medical Press. (**B**) Nanoparticles loaded with Pdgfb siRNA specifically reduced lung macrophage Pdgfb and prevented hypoxia-induced distal muscularization. Reproduced with permission [75]. Copyright. 2021, American Society for Clinical Investigation

Extracellular vesicles represent endogenous nanoscale materials whose distinctive physicochemical characteristics enable them to function as versatile drug delivery vehicles [173]. Early research investigated the role of MSC-Exos in HAPH, with a focus on macrophage targeting. MSC-Exos, but not fibroblast-derived exosomes, suppressed hypoxia-induced pulmonary macrophage influx, as evidenced by decreased macrophage numbers in bronchoalveolar lavage fluid. MSC-Exos blunted hypoxia-induced upregulation of pro-inflammatory mediators (MCP-1, HIMF) in bronchoalveolar lavage fluid, which are critical for macrophage recruitment and activation. Mechanistically, MSC-Exos suppressed hypoxia-induced STAT3 phosphorylation in the lung, a pathway involved in macrophage-mediated inflammation. Repeated MSC-Exos administration attenuated vascular remodeling, right ventricular hypertrophy, and HAPH development. These findings indicate MSC-Exos exert protective effects in HAPH by targeting macrophage-driven pulmonary inflammation, highlighting MSC-Exos as a potential therapeutic tool for regulating macrophage function in HAPH [174]. Another research explored the role of tadalafil-pretreated MSC-Exos in HAPH, with a focus on targeting macrophages. Tadalafil-pretreated MSC-Exos significantly attenuated lipopolysaccharide-induced inflammation in macrophages, reducing levels of pro-inflammatory factors (IL-6, TNF-α, IL-1β) more effectively than untreated MSC-Exos. Mechanistically, tadalafil-pretreated MSC-Exos upregulated miR-29a-3p expression via cAMP-responsive element binding protein 1 activation. MiR-29a-3p was transferred to macrophages through exosomes, directly targeting and functionally blocking ectonucleotide pyrophosphatase/phosphodiesterase 2 to exert anti-inflammatory effects. Depletion of miR-29a-3p in tadalafil-pretreated MSC-Exos abrogated these anti-inflammatory properties. In hypoxia-induced rat model, tadalafil-pretreated MSC-Exos targeted pulmonary macrophages, alleviating vascular remodeling and right ventricular dysfunction. These findings highlight that tadalafil-pretreated MSC-Exos enhances therapeutic efficacy in HAPH by targeting macrophages through the miR-29a-3p [175] (Fig. 14). More recent studies further elucidate the multifaceted therapeutic effects of human umbilical cord MSC-Exos, which promote macrophage M2 polarization while suppressing the IL-33/ST2 pathway, this dual modulation enhances anti-inflammatory IL-10 secretion and concurrently curbs PASMCs proliferation, ultimately ameliorating HAPH progression [176, 177].

The nanoparticle loading strategy provides a precise approach for macrophage-targeted therapy in HAPH. Aglaia Ntokou et al. developed nanoparticles carrying PDGF-B siRNA, which specifically downregulated PDGF-B expression in pulmonary macrophages of mice, thereby preventing hypoxia-induced distal muscularization, pulmonary hypertension, and right ventricular hypertrophy. Notably, biweekly intratracheal administration of nanoparticles for three consecutive weeks neither altered pulmonary mechanics and histological features nor resulted in off-target uptake by other organs such as the heart or liver [75].

Taken together, these findings underscore the therapeutic potential of macrophage-targeted strategies in HAPH, achieved through a variety of nanoscale delivery systems including lipid-based carriers, extracellular vesicles, and engineered nanoparticles. By leveraging these platforms, researchers have provided preclinical evidence supporting the attenuation of macrophage-mediated inflammation, vascular remodeling, and right ventricular dysfunction.

**Table 3:** Nanoplatform-based targeted therapy for HAPH

Names	Targets	Mechanisms	Refs
MSC-Exos	PASMCs	Hsp90aa1↓, ERK↓, pERK↓	[128]
MSC-Exos	PASMCs	YAP1↓, SPP1↓	[129]
MSC-Exos	PASMCs	EGFR↓, ERBB2↓	[130]
ITGB1-Exo	PASMCs	miR-429-3p↓, Rac1↓	[132]
212-eEV	PASMCs	Cell proliferation↓	[136]
GA-EVs	PASMCs	Cell proliferation↓	[137]
LVFF	RAGE	ACEI↓, RAGE↓	[139]
SA-PEG_2000_-NH_2_	PAECs, PASMCs	E-selectin↓	[140]
BPS-NP	PAECs, PASMCs	Vascular remodeling↓	[142]
NO-RP	PAECs	NO↑	[147]
mTOR siRNA/Ac-aCD NV	PASMCs	mTOR↓	[148]
PGC-1α nanoparticles	PAECs	PGC-1α↑	[144]
ACE2-CS-PRT@PM	PASMCs	ACE2↑	[145]
MOF_Cu_@WZ35	PASMCs	Cell proliferation and apoptosis↓	[151]
AuCeNPs	PASMCs	ROS↓	[152]
Sil@nanomil-89	PASMCs	Vasoconstriction↓	[153]
NP-siPdgfb	Macrophages	Pdgfb mRNA↓	[75]
MSC-Exos	Macrophages	MCP-1↓, HIMF↓, miR-17↓, miR-204↑, STAT3↓	[174]
MSC^TAD−Exo^	Macrophages	miR-29a-3p↑	[175]
MSC-Exos	Macrophages	IL-33↓, ST2↓, STAT3↓	[176, 177]

EGFR, epidermal growth factor receptor; ERBB2, Erb-B2 receptor tyrosine kinase 2; MSC-Exos, mesenchymal stromal cell-derived exosomes; LVFF, RAGE-targeted nanofibers containing the targeting epitope LVFFAED

## Discussion and perspective

Oxygen is crucial for cellular metabolism [178–180]. Low-pressure hypoxia at high altitudes exerts unique air pressure and environmental stress on lung immune cells, these stressors may regulate the phenotype, metabolic status and interaction with vascular cells of macrophages in a regionally specific manner, thereby making them coordinators of disease evolution and targets for treatment [72, 181]. HAPH is a progressive disorder driven by chronic hypobaric hypoxia, in which the immune system plays a pivotal role in disease pathogenesis and progression. Current research indicates that both AMs and IMs undergo significant hypoxia-induced alterations, including recruitment dynamics, M1/M2 polarization shifts, inflammatory activation, and metabolic reprogramming. These changes make macrophages both central coordinators of HAPH pathogenesis and attractive therapeutic targets. Importantly, recent advances in nanotechnology have expanded the therapeutic landscape by offering tools capable of precise macrophage modulation, pulmonary retention, and controlled drug release, thus providing new opportunities for disease intervention. In the future, nanoplatforms and immunotherapy targeting macrophages are expected to become key solutions for HAPH.

Macrophage recruitment represents a hallmark feature of HAPH, with increased cell numbers serving as the foundation for their subsequent functional roles. Macrophages exhibit a strong tendency to accumulate in hypoxic regions of tissues [72, 182], studies indicate that lung-resident macrophages can recruit circulating monocytes, either by attracting exogenous macrophages or by promoting their differentiation into functional macrophages [103]. Notably, this recruitment process may initially serve a protective role in hypoxia adaptation. Additionally, in vitro experiments have reported that hypoxia exposure (24 h) upregulates the expression of the anti-inflammatory cytokine IL-10 at the transcriptional level [72]. However, given that HAPH is a chronic and progressive disease, sustained macrophage recruitment may ultimately contribute to pathological remodeling, prolonged macrophage activation and infiltration may shift from adaptation to pathogenic remodeling [183]. Thus, targeting macrophage recruitment could represent a viable therapeutic strategy to slow disease progression, and the targeting capacity of nanocarriers offers a novel platform to specifically modulate this process while minimizing systemic side effects.

Macrophage polarization, a key feature of macrophage plasticity, enables functional diversification through differentiation into distinct subtypes. Nanotechnology offers the ability to deliver small molecules, siRNA or miRNA [184, 185] [186], which can highly specifically reprogram the polarization state of macrophages. Hypoxia-induced pro-inflammatory transformation and metabolic reprogramming represent critical phenotypic shifts in macrophages during HAPH pathogenesis [72]. HIF-1α serves as a master regulator in this process, orchestrating macrophage immune responses, survival, migration, phenotypic plasticity, and metabolic adaptation [187]. Under hypoxic conditions, elevated HIF activity drives profound metabolic alterations in macrophages, ultimately reshaping their phenotype and functional capabilities. HIF also mediates intercellular crosstalk between macrophages and other cell types within the pulmonary vascular microenvironment. Given its central role in these pathogenic mechanisms, HIF emerges as a promising therapeutic target for HAPH intervention [20, 188, 189]. Notably, nanoplatform-based immunomodulatory approaches to HIF regulation have already been explored in prior studies, suggesting that their application could be further extended to HAPH [190].

Furthermore, although AMs and IMs share similar pathogenic mechanisms in HAPH, their functional emphases differ, largely due to the distinct tissue microenvironments in which they reside, as the local milieu profoundly shapes macrophage phenotypes and functions [191]. AMs, located within the alveolar lumen and in direct contact with inhaled gases, undergo pro-inflammatory activation as a core adaptive response to hypoxia. Early recruitment and alternative activation of AMs play a critical role in shaping the later stages of HAPH progression [192]. By contrast, IMs exhibit extensive and sustained crosstalk with surrounding structural and vascular cells throughout both early and late disease stages. In addition to pro-inflammatory activation, IMs display marked hypoxia-induced metabolic reprogramming, which, together with aberrant intercellular interactions, sustains chronic inflammation and drives vascular remodeling, thereby contributing to the persistence and progression of HAPH. Moreover, differences in ontogeny may also underlie the functional heterogeneity of macrophages, for instance, macrophages derived from yolk sac possess strong proliferative and anti-apoptotic capacities [193, 194]. Intercellular communication between macrophages and lung-resident structural cells, particularly PAECs, PASMCs, and adventitial fibroblasts, constitutes a critical regulatory network in HAPH. Overall, AMs primarily regulate PAECs and PASMCs via HIF-1α and inflammatory mediators, thereby fostering a pulmonary microenvironment that is pro-inflammatory, pro-proliferative, and pro-migratory. In contrast, IMs exhibit more complex and integrative regulatory behavior, forming a dense crosstalk network with vascular cells. IMs-PAECs interactions are predominantly centered on inflammation-related pathways, while in IMs-PASMCs interactions, HIF-1α and IL-6 also play central roles but are more directly associated with PASMCs phenotypic changes, including proliferation, migration, and contraction. In addition, hypoxia-induced metabolic reprogramming in adventitial fibroblasts have emerged as key upstream triggers of macrophage activation, initiating both inflammatory and metabolic reprogramming in IMs. Thus, these interactions form a pathological network that drives vascular remodeling centered on macrophages. Nanomedicine can intervene by selectively modulating signaling pathways within this network. For example, intratracheal administration of liposomes encapsulating Bcar3 siRNA enables precise delivery of gene therapy agents to pulmonary macrophages and fibroblasts, thereby disrupting their crosstalk and attenuating the progression of idiopathic pulmonary fibrosis [195]. Therefore, it is reasonable to apply this technology to HAPH therapy, aiming to block hypoxia-induced pathological activation of macrophages and their crosstalk with pulmonary vascular cells.

Although existing research has provided strong insights into the pathogenesis of HAPH involving macrophages and immunotherapy targeting macrophages based on nanoplatforms, this research field is still in its infancy and requires further comprehensive studies.

Macrophages cannot be simply classified into AMs, IMs or M1/M2 macrophages. Macrophages have many subtypes [196]. Techniques such as single-cell sequencing, spatial omics, and lineage tracing have gradually revealed the dynamic changes and different subtypes of macrophages in time and space [51, 197, 198]. In addition, there is complex intercellular communication between macrophages and cells in the pulmonary microenvironment, especially between macrophages and PAECs, PASMCs and fibroblasts. These cells are the direct participants in pulmonary vascular remodeling. Revealing the interactions among them is conducive to finding key therapeutic targets [199].

**Fig. 15:**
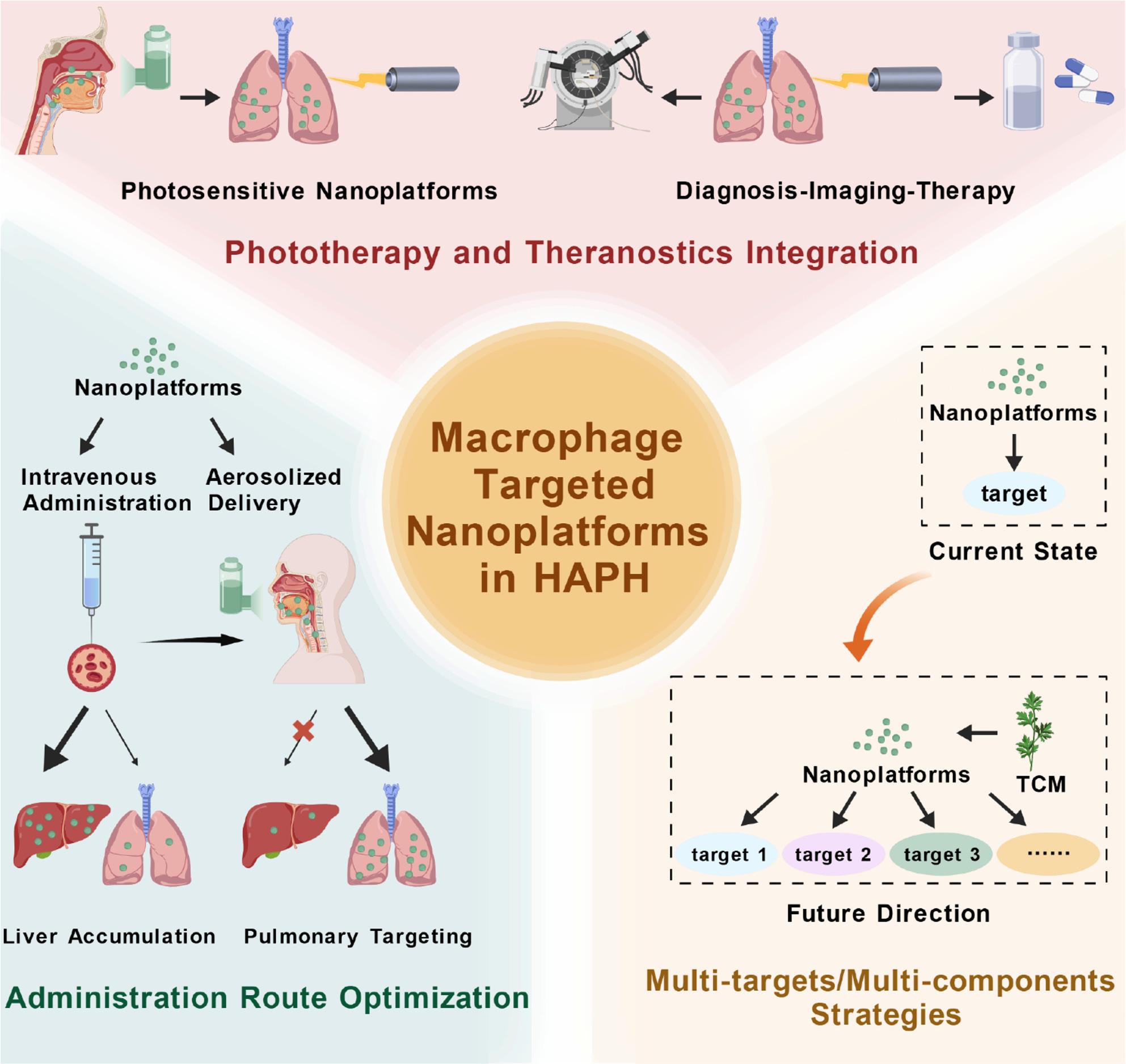
Developmental directions of macrophage-targeted nanoplatforms in HAPH. Centered on inhalable formulations, this strategy leverages natural multi-component, multi-target agents derived from traditional Chinese medicine, integrates phototherapy technologies, and enables dynamic macrophage modulation, ultimately advancing toward a comprehensive platform for “diagnosis-imaging-therapy” in HAPH (Created with BioGDP.com)

The incorporation of nanomaterial-based strategies into HAPH therapy represents a paradigm shift from symptomatic vasodilation to mechanistic precision intervention. Compared with conventional pharmacological approaches, these systems not only enhance drug bioavailability and pulmonary retention but also enable selective regulation of pathological cell populations, including macrophages, PAECs, and PASMCs. Nevertheless, several challenges remain before clinical translation. The route of administration has a profound impact on the biodistribution of nanocarriers. Most nanoplatforms undergo substantial hepatic and splenic clearance through the reticuloendothelial system, which compromises pulmonary delivery efficiency and may lead to off-target accumulation [200]. Studies have shown that following intravenous administration, up to 99% of injected nanoparticles accumulate in the liver due to systemic circulation clearance [201]. In contrast, aerosolized delivery leverages the unique physiological characteristics of the respiratory system, thereby enhancing pulmonary targeting while simultaneously improving patient quality of life. Therefore, in the context of HAPH, further development of aerosolized nanomedicine formulations holds great therapeutic promise. Furthermore, nanomaterials possess known immune-activating properties and thrombogenicity, and immune cells in the blood (such as monocytes, platelets, leukocytes, and dendritic cells) and tissues (such as resident phagocytes) tend to engulf and clear certain nanoparticles. The interaction of nanoparticles with plasma proteins (opsonins) and blood components (through hemolysis, thrombosis, and complement activation) may affect their uptake and clearance, potentially influencing their distribution and delivery to target sites [202]. Fortunately, this compatibility with the immune system largely depends on their surface chemistry, by altering the surface properties of nanomaterials, it is hoped that these side effects can be mitigated as much as possible [203, 204]. At present, most nanomaterial-based therapies focus on single targets. However, given the complex pathogenesis of HAPH, which involves multiple cell types and signaling pathways, future nanotherapeutic designs should integrate strategies capable of simultaneously modulating macrophages and other pathological components. In this regard, traditional Chinese medicine, with its multi-component and multi-target properties, offers a valuable reference. The natural phytochemical combinations in traditional Chinese medicine provide a holistic therapeutic approach that could inspire the development of next-generation nanomedicines with multi-target and multi-level regulatory capacities [205]. In recent years, phototherapy technologies have advanced rapidly and have been increasingly recognized for their potential in disease diagnosis and treatment due to their non-invasive nature, low risk of drug resistance, and precise targeting capabilities [206]. For example, ultrasmall gold nanoparticles exploiting NIR-II luminescence have shown promise across a range of biomedical applications, including imaging and therapeutic intervention [207]. In the context of HAPH, given that pathological changes are primarily confined to small pulmonary arteries and surrounding tissues, and that photosensitive nanoparticles can be delivered via the respiratory route, phototherapy holds promise for achieving precise immunomodulation through biomarker-based diagnosis and subtype-specific targeting of macrophages, thereby enabling an integrated “diagnosis-imaging-therapy” approach [206, 208] (Fig. 15).

## Conclusion

In conclusion, macrophages play a central and multifaceted role in the pathogenesis of HAPH. Leveraging nanomaterial-based platforms to precisely target macrophage recruitment, M1/M2 polarization, inflammatory activation, and metabolic reprogramming offers a promising avenue for mechanistic intervention. Integrating nanotechnology with macrophage-focused immunotherapies not only enhances delivery efficiency and tissue specificity but also enables combinatorial strategies, such as gene modulation, gas therapy, and phototherapy, for precise immunomodulation. Continued investigation into these integrated approaches may provide innovative and translational strategies for effective HAPH management.

## Data Availability

No datasets were generated or analysed during the current study.

## Abbreviations

ACE: Angiotensin-converting enzyme
AKT: Protein kinase B
AMs: Alveolar macrophages
β3AR: β3-adrenergic receptor
Bcr: Breakpoint cluster region
BMPR2: Bone morphogenetic protein receptor 2
CCL: C-C Motif chemokine ligand
CCR: C-C chemokine receptor
CtBP1: C-terminal binding protein 1
CSF-2: Colony-stimulating factor 2
CX3CL1: C-X3-C Motif chemokine ligand 1
CX3CR1: C-X3-C Motif chemokine receptor 1
CXCL: C-X-C Motif chemokine ligand
C/EBPβ: CCAAT/enhancer binding proteinβ
ERK: Extracellular signal-regulated kinase
ET-1: Endothelin-1
eNOS: Endothelial nitric oxide synthase
GM-CSF: Granulocyte macrophage colony-stimulating factor
Foxp1: Forkhead box p1
HAPH: High altitude pulmonary hypertension
HDAC: Histone deacetylase
HIF: Hypoxia-inducible factor
HIMF: Hypoxia-induced mitogenic factor
HMOX1: Heme oxygenase 1
Hsp90aa1: Heat shock protein 90 alpha family class A member 1
IL: Interleukin
IMs: Interstitial macrophages
iNOS: Inducible nitric oxide synthase
IRS2: Insulin receptor substrate 2
κ-OR: κ-opioid receptor
LOX-1: Low-density lipoprotein receptor-1
MAPK: Mitogen-activated protein kinase
MCP-1: Monocyte chemoattractant protein-1
MSC-Exos: Mesenchymal stromal cell-derived exosomes
mTOR: Mammalian target of rapamycin
MyD88: Myeloid differentiation primary response 88
NF-κB: Nuclear factor-kappa B
PAECs: Pulmonary artery endothelial cells
PASMCs: Pulmonary artery smooth muscle cells
PDGF: Platelet-derived growth factor
Peli1: Pellino E3 ubiquitin-protein ligase 1
PFKFB3: 6-phosphofructo-2-kinase/fructose-2,6-bisphosphatase 3
PGC-1α: Peroxisome proliferator-activated receptor gamma coactivator-1
PTEN: Phosphatase and tensin homolog
SDF-1α: Stromal cell-derived factor 1α
SGK1: Serum glucocorticoid-regulated kinase-1
SPP1: Secreted phosphoprotein 1
STAT: Signal transducer and activator of transcription
ST2: Suppression of tumorigenicity 2
Tfh: T follicular helper
TNF-α: Tumor necrosis factor-α
TSP-1: Thrombospondin-1
VEGF-A: Vascular endothelial growth factor A
VEGFR2: Vascular endothelial growth factor 2
YAP1: Yes associated protein 1
Ythdf2: YTH N6-methyladenosine RNA binding protein 2
